# Improved detection of common variants in coronary artery disease and blood pressure using a pleiotropy cFDR method

**DOI:** 10.1038/s41598-019-46808-2

**Published:** 2019-07-17

**Authors:** Xiang-Jie Mao, Qiang Zhang, Fei Xu, Pan Gao, Nan Sun, Bo Wang, Qi-Xin Tang, Yi-Bin Hao, Chang-Qing Sun

**Affiliations:** 1 College of Public Health, Zhengzhou University, 100 Kexue Avenue, Zhengzhou, 450001 Henan People’s Republic of China; 2 Department of Management Information Systems, Terry College of Business, University of Georgia, Athens, Georgia USA; 3 People’s Hospital of Zhengzhou, Zhengzhou, 450000 Henan People’s Republic of China

**Keywords:** Genome-wide association studies, Epidemiology

## Abstract

Plenty of genome-wide association studies (GWASs) have identified numerous single nucleotide polymorphisms (SNPs) for coronary artery disease (CAD) and blood pressure (BP). However, these SNPs only explain a small proportion of the heritability of two traits/diseases. Although high BP is a major risk factor for CAD, the genetic intercommunity between them remain largely unknown. To recognize novel loci associated with CAD and BP, a genetic-pleiotropy-informed conditional false discovery rate (cFDR) method was applied on two summary statistics of CAD and BP from existing GWASs. Stratified Q-Q and fold enrichment plots showed a high pleiotropic enrichment of SNPs associated with two traits. Adopting a cFDR of 0.05 as a threshold, 55 CAD-associated loci (25 variants being novel) and 47 BP loci (18 variants being novel) were identified, 25 of which were pleiotropic loci (13 variants being novel) for both traits. Among the 32 genes these 25 SNPs were annotated to, 20 genes were newly detected compared to previous GWASs. This study showed the cFDR approach could improve gene discovery by incorporating GWAS datasets of two related traits. These findings may provide novel understanding of etiology relationships between CAD and BP.

## Introduction

As one of the leading causes of human mortality and disability all over the world, coronary artery disease (CAD) is the most common heart disease characterized by the declining of arterial elastic properties and the deposition of lipid-rich atheroma^1,2^. Previous studies suggested that CAD was a complex multifactorial disease with both genetic and environmental determinants^3–5^. Heritability of CAD was estimated to be approximately 40% to 60%, which indicated that genetic determinants contribute significantly to the development of CAD^6^. However, the known CAD loci together only explained 8.53% of CAD heritability^7^. Systolic and diastolic blood pressure (SBP and DBP) are two common complex traits with high heritability and the related genetic variants can lead to hypertension^1,8,9^. Elevated BP is a major risk factor for cardiovascular diseases. To date, genome-wide association studies (GWASs) have identified more than 120 loci related to blood pressure (BP), and 107 independent loci were validated by Warren *et al*.^10^.

There have been substantial epidemiological evidences to demonstrate that BP is associated with risk of CAD^11–13^. A GWAS reported that BP risk score was positively associated with stroke (*P* = 6.0 × 10^−6^), alterations of cardiac structure (*P* = 3.3 × 10^−5^) and CAD (*P* = 8.1 × 10^−29^)^14^. Another study involving genetic risk scores based on 26 BP-associated single nucleotide polymorphisms (SNPs) showed that the SBP and DBP related risk alleles had 70% and 59% higher odds of increasing CAD, respectively^15^. Other evidences suggested that genetic pleiotropic effect exists in CAD and BP. Genetic pleiotropy is the phenomenon of a single gene or variant being related to two or more phenotypes^16–18^. A meta-analysis study reported that SNP rs12413409 for CAD was detected to be associated with hypertension^19^. Besides, a study in East Asian individuals discovered four SNPs (rs16849225, rs16998073, rs1173766, and rs2681472) for both BP and CAD^20^. These findings indicated that related traits may share common genetic mechanisms. Despite numerous various GWASs have been successfully applied in identifying large number of SNPs associated with CAD or BP, these SNPs only explain a small proportion of the heritability of two traits. Although GWAS studies may increase statistical power in larger samples, it is often not feasible since the traditional GWAS methods is too costly.

To explain a greater proportion of genetic mechanisms in the pathogenesis of CAD and BP, further innovative analytical methods are required to discover novel SNPs or genes, especially novel overlapped genetic variants. As a recently developed analytical method, the conditional false discovery rate (cFDR)^16–18^, only demands summary statistics results of independent GWAS datasets of correlated traits/diseases. Based on genetic pleiotropy, statistical power and identification of genetic loci will be greatly improved by incorporation two GWAS datasets. This method has been successfully applied to a number of diseases and phenotypes, including schizophrenia and bipolar disorder^16^, blood pressure and associated phenotypes^17^, and schizophrenia and cardiovascular-disease risk factors^18^. In addition, the cFDR has recently been applied by our group to the joint analysis of type 2 diabetes and birth weight^21^, height and femoral neck bone mineral density^22^, CAD and bone mineral density^23^.

In this study, to further exploring the genetic architecture and potential etiology of CAD and BP, the cFDR approach was utilized in two large and existing datasets^14,19^ for two traits to detect novel common variants and pleiotropic susceptibility loci. We hope to improve SNP detection by cFDR and obtain some novel insights into the unknown shared biological mechanisms between them.

## Results

### Estimation of pleiotropic enrichment

**Figure 1:**
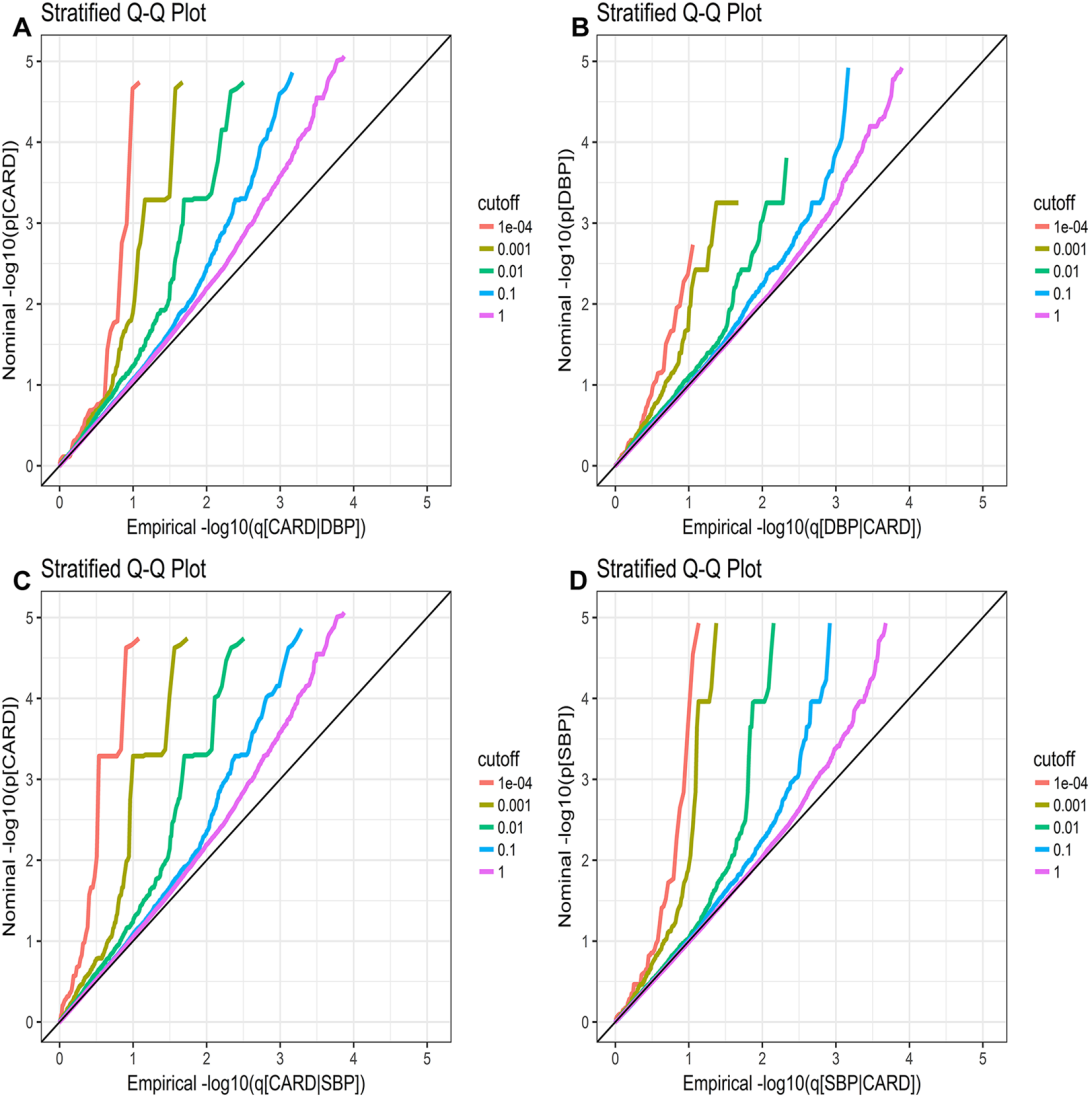
Stratified Q–Q plots of discovery analysis. Stratified Q-Q plot of nominal versus empirical log_10_ *P*-values (corrected for inflation) in (**A)** CAD below the standard GWAS threshold of *P* = 5 × 10^−8^ as a function of significance of the association with DBP at the level of −log_10_(*P*) > 0, −log_10_(*P*) > 1, −log_10_(*P*) > 2, −log_10_(*P*) > 3, and −log_10_(*P*) > 4 corresponding to *P* < 1, *P* < 0.1, *P* < 0.01, *P* < 0.001, and *P* < 0.0001, respectively, and in (**B)** DBP below the standard GWAS threshold of *p* = 5 × 10^−8^ as a function of significance of association with CAD, and in **C)** CAD below the standard GWAS threshold of *P* = 5 × 10^−8^ as a function of significance of association with SBP and in (**D)** SBP below the standard GWAS threshold of *P* = 5 × 10^−8^ as a function of significance of association with CAD. Black solid lines indicate the null-hypothesis.

A significant pleiotropic enrichment was shown in stratified Q-Q (Fig. 1) and TDR plots (Fig. S1). As reflected in Fig. 1A for CAD conditioned on DBP, the great spacing (leftward shifts) between different stratified Q-Q curves indicated strong level of enrichment and great proportion of true associations for any given CAD nominal *P*-values. The conditional Q-Q plot for DBP conditional on CAD (Fig. 1B) showed some pleiotropic enrichment across various levels of significance for CAD. In Fig. 1C,D, similar results with Fig. 1A were obtained.

**Figure 2:**
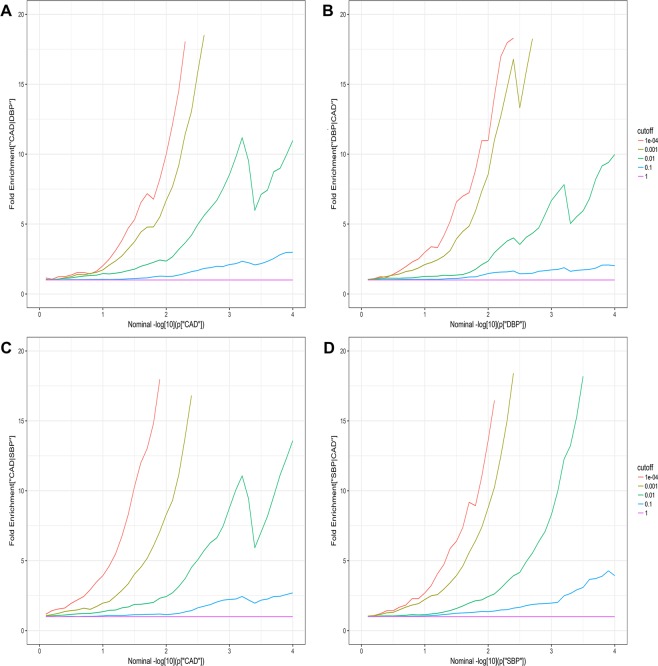
Fold-enrichment plots of discovery analysis. Fold-enrichment plots of enrichment versus nominal −log_10_ *P*-values in (**A)** CAD below the standard GWAS threshold of *P* < 5 × 10^−8^ as a function of significance of the association with DBP, and in (**B)** DBP below the standard GWAS threshold of *P* < 5 × 10^−8^ as a function of significance of the association with CAD, and in (**C)** CAD below the standard GWAS threshold of *P* < 5 × 10^−8^ as a function of significance of the association with SBP and in (**D)** SBP below the standard GWAS threshold of *P* < 5 × 10^−8^ as a function of significance of the association with CAD. The purple line with slope of zero represents all SNPs.

Based on the fold-enrichment plots, we observed approximately an 18-fold increase for CAD and DBP (Fig. 2A,B) in the proportion of SNPs reaching the genome wide significance level of −log_10_(*P*) > 7.3 when comparing the subset with the most stringent conditional association (*P* = 1 × 10^−4^) to the group with all SNPs (*P* = 1). An 18-fold increase was also observed for CAD conditional on SBP (Fig. 2C). In Fig. 2D, about 16-fold increase was observed for SBP.

### CAD loci identified with cFDR

Based on the enrichment of pleiotropic effect between CAD and BP in step one, we performed the cFDR analysis on them to investigate which variants were related to CAD and BP.

Conditional on their association with DBP, 42 SNPs associated with CAD were detected (Table S2 and Fig. S2A) with cFDR < 0.05, which were located on 14 chromosomes. Conditional on their association with SBP, 44 SNPs for CAD were discovered (Table S3 and Fig. S2B), which were mapped to 15 different chromosomes. Total of 55 independent SNPs (which were annotated to 67 genes) related to CAD were identified. Ten loci (rs964184, rs10774625, rs10744777, rs9515203, rs4773144, rs11617955, rs17514846, rs2252641, rs7651039 and rs9381462) of these SNPs reached genome-wide significance at 5 × 10^−8^ in the original and previous CAD related GWASs (Table S4)^7,19,24^. 20 SNPs were in high linkage disequilibrium (LD) (R^2^ > 0.6) with other CAD-associated SNPs reported previously (Table S5) and the rest 25 novel SNPs were not previously reported in original CAD-related GWASs or any other CAD studies. For the 66 genes annotated by these SNPs, 28 genes were previously reported in CAD GWASs^7,24–26^. Among all the 55 independent loci for CAD, most of the genes were enriched in CAD-related terms such as “multicellular organism development”, “response to growth factor” and “organelle lumen”. Detailed information of GO term enrichment analysis was shown in Table 1.

**Table 1:** Functional Term Enrichment Analysis.

Pathway ID	Pathway description	Count in gene set	*P*-value
CAD GO:0043233	organelle lumen	26	7.58 × 10^−5^
GO:0031974	membrane-enclosed lumen	26	7.58 × 10^−5^
GO:0007275	multicellular organism development	25	9.55 × 10^−5^
GO:0070848	response to growth factor	11	4.33 × 10^−8^
GO:0060976	coronary vasculature development	2	5.48 × 10^−3^
GO:0060977	coronary vasculature morphogenesis	2	8.22 × 10^−4^
GO:0007166	cell surface receptor signaling pathway	12	1.23 × 10^−2^
BP GO:0007596	blood coagulation	3	3.89 × 10^−2^
GO:0072359	circulatory system development	9	1.80 × 10^−3^
GO:0031323	regulation of cellular metabolic process	26	3.60 × 10^−3^
GO:0035556	intracellular signal transduction	12	5.49 × 10^−3^
GO:0007155	cell adhesion	6	2.23 × 10^−2^
GO:0035556	intracellular signal transduction	12	5.49 × 10^−4^
GO:0048514	blood vessel morphogenesis	5	2.95 × 10^−3^
GO:0072358	cardiovascular system development	6	1.63 × 10^−3^
GO:0071363	cellular response to growth factor stimulus	7	1.81 × 10^−4^
GO:0051173	positive regulation of nitrogen compound metabolic process	20	1.56 × 10^−5^
GO:0005515	protein binding	37	3.72 × 10^−2^
CAD&BP GO:0031091	platelet alpha granule	2	6.30 × 10^−3^
GO:0070851	growth factor receptor binding	3	7.32 × 10^−4^
GO:0007154	cell communication	14	3.80 × 10^−3^
GO:0050789	regulation of biological process	22	5.73 × 10^−3^
GO:0051128	regulation of cellular component organization	10	3.91 × 10^−4^
GO:0070848	response to growth factor	6	3.47 × 10^−5^
GO:0008015	blood circulation	5	1.10 × 10^−4^
GO:0009893	positive regulation of metabolic process	13	7.14 × 10^−5^

### BP loci identified with cFDR

We detected 28 SNPs associated with DBP given their association with CAD (Table S6 and Fig. S2C), which were located on 10 chromosomes. And 33 SNPs for SBP were discovered (Table S7 and Fig. S2D), which were mapped to 15 different chromosomes. Total of 47 independent BP-SNPs (which were annotated to 66 genes) were identified (Table S8). Eleven SNPs were previously reported associated with SBP/DBP in diverse ancestry^27,28^. 18 SNPs were in high LD (R^2^ > 0.6) with other BP-associated SNPs reported previously (Table S9) and the rest 18 novel SNPs were not reported in the previous BP-related GWASs or any other BP studies. For the 67genes annotated by these SNPs, 32 of these genes were previously reported for BP in GWASs^10,27–32^. Among the 47 BP-related loci, some of the genes were enriched in BP-related terms such as “circulatory system development”, “regulation of cellular metabolic process” and “protein binding”. Detailed information of GO term analysis was shown in Table 1.

### Pleiotropic loci for both CAD and BP

**Figure 3:**
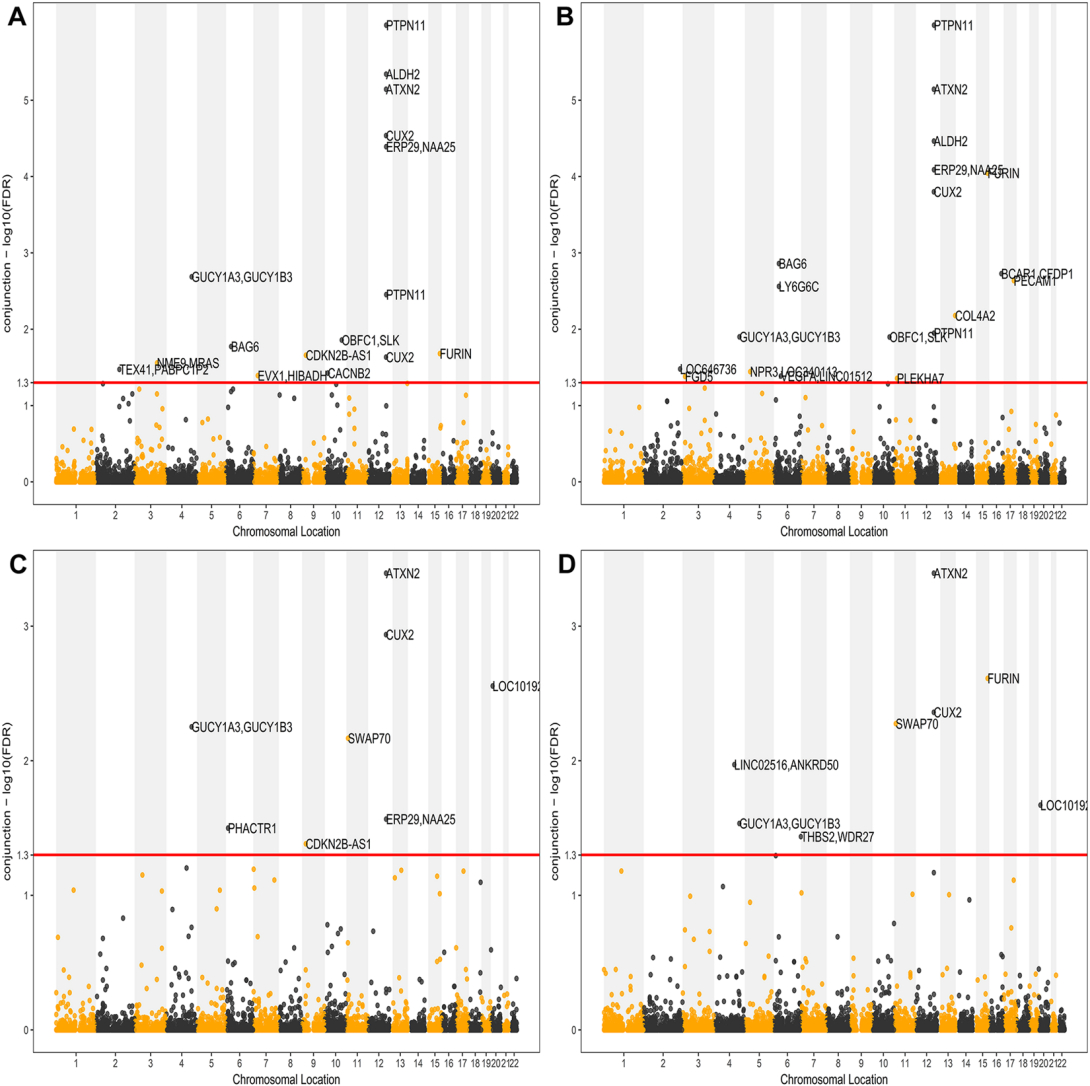
“Conjunctional Manhattan plot” of conjunctional −log_10_ (cFDR) values for CAD and BP. Pleiotropic SNPs with conjunctional −log_10_ cFDR > 1.3 (i.e. ccFDR < 0.05) are shown above the red line. Upper Panel: conjunctional Manhattan plot for CAD and DBP (in **A**), and conjunctional Manhattan plot for CAD and SBP (in **B**). Details for all significant loci are given in Table S10 and Table S11. Lower Panel: The plots showed conjunctional Manhattan plots for CAD and DBP/SBP (**C**,**D**) in the C4D dataset.

The ccFDR analysis detected 16 pleiotropic SNPs that were associated with both CAD and DBP (Fig. 3A and Table S10). And 19 pleiotropic SNPs related to both CAD and SBP were detected (Fig. 3B and Table S11). Total of 25 independent pleiotropic SNPs associated with both CAD and BP were identified (Table 2). 12 of 25 SNPs were confirmed to be related to both traits and other 13 SNPs were novel pleiotropic variants. Four SNPs of which (rs7902587, rs10744777, rs4678408 and rs998584) were reported to be associated with thyroid cancer, ischemic stroke, type 2 diabetes and body mass index in previous GWASs^31,33–36^. For the 32 genes the detected pleiotropic SNPs were annotated to, 12 genes (*SLK*, *PLEKHA7*, *ATXN2*, *CUX2*, *COL4A2*, *FURIN*, *CFDP1*, *TEX41*, *FGD5*, *MRAS*, *VEGFA*, *CDKN2B-AS1*) were previously reported for both traits^10,19,24,28,29^. Most of the pleiotropic SNPs were resided in the intronic (60%) and intergenic (36%) regions while only one was located in the untranslated regions (4%). Of the detected 25 pleiotropic loci, most of the genes were enriched in CAD and BP related terms such as “cell communication”, “response to growth factor”, and “positive regulation of metabolic process”. Detailed information of GO term analysis was shown in Table 1.

**Table 2:** Conjunctional cFDR: pleiotropic loci in CAD and BP.

CHR	RSID	Gene	Role	Discovery analysis			Replication analysis		SNP type
				P.CAD	P.BP	ccFDR	SNP	ccFDR	
chr10	rs7902587	*OBFC1*, ***SLK***^**a**^	intergenic	7.07E-05	2.25E-03	1.27E-02			Novel
chr10	rs7069531	*CACNB2*	intronic	2.18E-03	7.21E-04	3.76E-02			Novel
chr11	rs366590	***PLEKHA7*** ^**a**^	intronic	1.60E-02	3.78E-06	4.40E-02			Novel
chr12	**rs11066301** ^**b**^	*PTPN11*	intronic	5.20E-07	4.94E-08	1.04E-06			CAD/DBP
chr12	**rs10774625** ^**b**^	***ATXN2*** ^**a**,^ *	intronic	7.19E-06	1.13E-09	7.19E-06	rs653178, R2 = 0.9108	4.04E-04	CAD/BP
chr12	**rs10744777** ^**b**^	*ALDH2*	intronic	1.52E-06	6.24E-06	3.43E-05			CAD/DBP
chr12	**rs4767293** ^**b**^	ERP29*, NAA25*	intergenic	1.81E-05	7.98E-06	8.15E-05	rs4767293	2.71E-02	CAD/DBP
chr12	**rs7970490** ^**b**^	***CUX2*** ^**a**,^ *	intronic	2.18E-05	2.83E-05	1.58E-04	rs7970490	1.15E-03	CAD/DBP
chr12	**rs11066322** ^**b**^	*PTPN11*	intronic	1.76E-03	2.25E-04	1.13E-02			CAD/BP
chr12	**rs6489979** ^**b**^	***CUX2*** ^**a**,^ *	intronic	1.71E-04	2.03E-03	2.32E-02			CAD/BP
chr13	rs9515203	***COL4A2*** ^**a**^	intronic	3.42E-05	1.45E-03	6.63E-03			Novel
chr15	**rs17514846** ^**b**^	***FURIN*** ^**a**,^ *	intronic	2.37E-05	1.17E-05	9.01E-05	rs17514846	2.44E-03	CAD/SBP
chr16	**rs4243111** ^**b**^	*BCAR1*, ***CFDP1***^**a**^	intergenic	9.27E-05	2.26E-04	1.87E-03			CAD/SBP
chr17	**rs2812** ^**b**^	*PECAM1*	UTR3	4.25E-04	7.43E-05	2.31E-03			CAD
chr2	rs6713510	*LOC646736*	ncRNA_intronic	9.77E-05	5.44E-03	3.32E-02			Novel
chr2	rs16824790	***TEX41***^**a**^, *PABPC1P2*	intergenic	6.97E-05	5.34E-03	3.36E-02			Novel
chr3	rs13070927	***FGD5*** ^**a**^	intronic	9.95E-03	1.13E-04	4.16E-02			Novel
chr3	rs4678408	*NME9*, *MRAS*^a^	intergenic	4.69E-04	1.00E-03	2.77E-02			Novel
chr4	**rs7698460** ^**b**^	GUCY1A3*, GUCY1B3*	intergenic	1.03E-03	5.09E-04	1.26E-02			CAD/BP
chr5	rs13154066	*NPR3*, *LOC340113*	intergenic	2.69E-02	2.12E-07	3.59E-02			Novel
chr6	**rs1077393** ^**b**^	*BAG6*	intronic	5.17E-04	2.22E-06	1.38E-03			CAD/BP
chr6	rs805293	*LY6G6C*	intronic	5.00E-04	1.09E-04	2.75E-03			Novel
chr6	rs998584	***VEGFA***^**a**^, *LINC01512*	intergenic	9.02E-03	9.30E-05	4.11E-02			Novel
chr7	rs4722680	*EVX1*, *HIBADH*	intergenic	2.18E-02	5.48E-06	4.05E-02			Novel
chr9	rs10965212	***CDKN2B-AS1*** ^**a**,^ *	ncRNA_intronic	1.37E-17	2.19E-02	2.19E-02	rs7049105, R2 = 0.99602	4.14E-02	Novel

**Notes:** The R^2^ is the measure of linkage disequilibrium (LD) between the identified SNP and the SNP which is significant in the replication analysis or CAD/BP related studies. If the R^2^ value is greater than 0.6, it represents that these two SNPs are in high LD, this SNP is considered to be replicated/reported.

^**a**^Genes identified in our study have been reported to be associated with both CAD and BP in original and previous GWAS studies.

^**b**^**SNP type** means whether pleiotropic SNPs identified in our study to be associated with both CAD and BP.

^*^Pleiotropic genes identified in discovery analysis further confirmed in the replication analysis.

**CHR:** chromosome, **RSID:** SNP ID (rs number), **ccFDR:** Conjunctional conditional false discovery rate, **CAD:** coronary artery disease, **SBP:** systolic blood pressure, **BP:** blood pressure.

### Replication analysis

To address the possibility that the observed pattern of enrichment may result from spurious associations, we performed a replication analysis (Tables S12–S14). First, we observed a similar pleiotropic enrichment pattern by the stratified Q-Q plots in replication analysis (Fig. S3). In the discovery phase of analysis, we detected 55 and 47 variants associated with CAD and BP, respectively. In replication analysis, we replicated 7 and 15 variants associated with CAD and BP, respectively (Table S4 and Table S8). For the pleiotropic loci which related to both traits, 5 SNPs and 8 genes were replicated (Table 2, Fig. 3C,D). These results showed that the pleiotropic enrichment between BP and CAD was largely consistent and some common variants can be replicated across studies.

## Discussion

By applying the cFDR approach on GWAS summary statistics of CAD and BP, we found and replicated the enrichment of pleiotropic effect between CAD and BP. Combining these two CAD and BP GWAS samples could improve identification of common variants associated with two phenotypes by increasing statistical power. Andreassen *et al*.’s study demonstrated the cFDR resulted of the number of SNPs can in an increase of 15–20 times. Using traditional FDR methods in the separate GWAS studies, 25 and 29 genetic variants were discovered for CAD and BP, respectively. Adopting the pleiotropy-informed cFDR method, we identified a total of 55 CAD susceptibility SNPs and 47 SNPs in BP, among of them 30 CAD-associated SNPs and 29 BP-associated SNPs were verification in the original or previous CAD/BP-related studies. Moreover, this method enables identification of shared loci associated with both CAD and BP by leveraging the pleiotropic polygenic effects. Total of 25 pleiotropic SNPs (which were annotated to 32 genes) were discovered through ccFDR analysis, among which 13 were novel.

The novel findings may lead us to a better understanding of the overlapping genetic mechanisms and common etiology between these related traits in different gene regions. Seven novel pleiotropic genes including *NME9*, *NPR3*, *BAG6*, *CACNB2*, *PTPN11*, *HIBADH* and *BCAR1* were all related to SBP and DBP in previous GWASs^28,30–32^. *PABPC1P2* was associated with schizophrenia and *OBFC1* as a locus involved in human leukocyte telomere biology in previous GWASs^37,38^. *ALDH2* and *EVX1* were associated with interaction of SBP and alcohol consumption^39^. Six novel pleiotropic genes (*LINC01512*, *LY6G6C*, *LOC340113*, *GUCY1B3*, *GUCY1A3* and *LOC646736*) were not reported in any diseases/traits GWASs previously. As examples, we will discuss two of these genes *PECAM1* and *ERP29* for their potential functional relevance.

The pleiotropic SNP rs2812 was located in the untranslated region (UTR) of platelet endothelial cell adhesion molecule-1 gene (*PECAM1*), which was associated with CAD in GWAS consisting of both European and South Asians population^25^. The knockdown of *PECAM1* in a mice model could reduce cell-cell contacts, which suggested *PECAM1* participated in regulation of flow-stimulated Gab1 (Grb2-associated binder-1) tyrosine phosphorylation and signal transduction of cell by Gab1-eNOS pathway^40^. In another study, Gab1 tyrosine phosphorylation exerted a key role in promoting angiogenesis and regulating endothelial nitric oxide (NO) synthase (eNOS) activation^41^. Moreover, endothelial cells (ECs) were determinants of inflammation and some enhancers in ECs are related to CAD. Dynamic endothelial enhancer elements improved understanding of vascular inflammatory diseases^42^. The eNOS inactivation is an important characterize of endothelial dysfunction. Endothelial dysfunction is a common mechanism that can lead to several cardiovascular diseases, including atherosclerosis, CAD and hypertension^43–45^. Taken together, *PECAM1* may contribute to the development of CAD and BP via PECAM1-Gab1-eNOS pathway.

SNP rs4767293 was located in the intergenic region between *NAA25* and endoplasmic reticulum protein 29 gene (*ERP29*). *NAA25* and *ERP29* were associated with inflammatory bowel diseases (IBDs), which include Crohn’s disease and ulcerative colitis^46^. Several epidemiology studies suggested that IBDs were potential risk factors for cardiovascular diseases^47–49^. Additionally, IBDs are chronic inflammatory diseases, later stage of which could contribute to endothelial dysfunction and platelet aggregation in artery blood vessels^50^. *ERP29* was localized in the endoplasmic reticulum (ER) and expressed among various tissues and cell types that included N-terminal and C-terminal domains^51^. Furthermore, *ERP29* is a tumor suppressor gene via ERP29-MGMT (O^6^-methylguanine DNA-methyltransferase) axis to exert the function of radioresistant in MDA-MB-231 breast cancer cells^52^. *ERP29* was involved in the formation of epithelial cells by junction transmembrane proteins, and regulation of the epithelial–mesenchymal transition (EMT) in epithelial cells to influence cancer progression^53,54^. A recent study showed that pigment epithelium-derived factor by suppressing Wnt/β-catenin pathway to reduce endothelial cell injury so as to prevent the formation of atherosclerosis^55^. However, to our knowledge the relational pathways for IBD and CAD are still largely unknown, which required to further explore in future studies.

There are several advantages in this study. First, through the incorporation of two GWAS datasets expanded the sample size and increased the statistical power, which contributed to successful discovery of novel SNPs for CAD and BP. Second, both datasets were all European individuals in this study. We analyzed both two phenotypes novel genetic variants to improve understanding of genetic relationship in CAD and BP. The findings were also partially validated by GO terms analysis and some variants were also further replicated to be associated with CAD or BP in the replication analysis. Third, we investigated and identified 25 shared common variants in CAD and BP (including SBP and DBP), while the etiology mechanisms of CAD and DBP were ignored in most previous studies. However, there are some limitations for this study. First, we did not replicate all the variants in C4D datasets, possibly due to the C4D dataset was derived from a meta-analysis of only four GWAS of European and South Asian descent. Second, some individuals were overlapped between two datasets, which might lead to increase of false positive rate. To minimize this error, the high LD of SNPs were considered to be replicated/reported. Third, the existing method of GWAS studies cannot be compared with the cFDR approach due to lack of the raw genotype and phenotype data for both traits.

In conclusion, this study showed the high availability of cFDR method in improving identification of genetic loci by incorporating two datasets of related traits. We found high pleiotropic enrichment between CAD and BP and identified several novel pleiotropic loci for both traits. The novel susceptibility loci may provide us novel implications in potential shared genetic mechanistic between these two phenotypes.

## Materials and Methods

### GWASs datasets

The GWAS datasets for CAD and BP were acquired from publicly available websites. The BP dataset was performed by the International Consortium for Blood Pressure Genome-Wide Association Studies (ICBP) and downloaded from https://www.nature.com/nature/journal/v478/n7367/full/nature10405.html#group-1. This GWAS meta-analysis contains association summary statistics for 69,395 individuals of European ancestry. Two CAD datasets were downloaded from http://www.cardiogramplusc4d.org/data-downloads/. The CARDIoGRAM dataset was performed by Coronary Artery Disease Genome-Wide Replication and Meta-Analysis consortium, which is a meta-analysis of 14 GWASs of CAD contains association summary statistics for European ancestry of 22,233 cases and 64,762 controls. The C4D dataset performed by the Coronary Artery Disease (C4D) Genetics Consortium was derived from a meta-analysis of four large GWAS of European and South Asian descent involving 15,420 cases and 15,062 controls. All datasets both provide summary statistics for each SNP and its corresponding *P*-value after adopting genomic control both at individual study level and after meta-analysis. Furthermore, the C4D dataset in our analysis was used as the replication dataset. More details about recruitment, phenotyping, genotyping and association analyses were described in the original GWASs publications^14,19,25^. Contributing studies received ethical approval from their respective institutional review boards.

### Data preparation

Before the analysis, we checked overlapping samples included in these datasets of the cohorts. We found 1,121 individuals were overlapped between CARDIoGRAM and ICBP datasets, and no overlapped individuals between CARDIoGRAM and C4D datasets (Table S1). Genomic control (GC) corrections has been applied in those original datasets at the individual study level and for the meta-analysis to ensure the variance estimation for each SNP would not be inflated due to population heterogeneity^56^.

### Statistical analysis

All cFDR analysis was performed in “GWAScFDR” packages of R software 3.43. The “ggplot2” and “Rmanhattanplot” packages were used to conduct stratified Q-Q plots, fold-enrichment plots and Manhattan plots. Using this approach, we obtained four look-up tables–cFDR results for CAD conditioned on SBP/DBP and vice versa. We identified loci associated with BP and CAD (cFDR < 0.05) using these tables.

### Stratified Q-Q and enrichment plots for pleiotropic enrichment estimation

The stratified Q-Q plot was used to assess the pleiotropic enrichment of genetic loci between both traits. The stratified Q-Q plots usually present the nominal *P*-value (−log_10_(*p*)) on the y-axis, denoted by “p” against the empirical quantiles (−log_10_(q)) on the x-axis, here denoted by “q”. Stratified Q–Q plots were constructed by nominal *P*-value of the principal trait SNPs conditional on SNPs associated with the second phenotype at varying levels. The pleiotropy enrichment can be seen from the degree of leftward shift from the expected null line as the principal trait is successively conditioned on different significance levels of the second phenotype. If pleiotropic enrichment does exist, an earlier leftward shift from the null line will be present. Greater spacing between stratified Q–Q curves visually indicates a higher level of pleiotropic enrichment between two traits. Pleiotropic enrichment can also be interpreted in terms of stratified true discovery rate (TDR) plots (equivalent to 1-FDR) (Fig. S1). Stratified TDR plots illustrating the increase in TDR associated with increased pleiotropic enrichment. The conservatively estimated FDR is directly related to the horizontal shift of the curves from the cut off line x = y in the stratified Q-Q plots, with a larger shift corresponding to a smaller FDR.

In order to check whether the pleiotropic effect enrichment was consistent, we conducted a replication analysis. CARDIoGRAM dataset for CAD was used as a discovery dataset for cFDR and conjunction FDR analyses with BP, the C4D dataset was independent of CARDIoGRAM for the replication analysis.

To confirm the enrichment effects, fold-enrichment plots were conducted. We present fold-enrichment plots of nominal −log_10_(*P*) values for CAD SNPs below the standard GWAS threshold of *P* < 5 × 10^−8^ and for subsets of SNPs determined by the significance of their association with DBP/SBP and *vice versa*. Fold-enrichment is assessed by the degree of upward shift from the null line.

### cFDR and conditional Manhattan plots

In order to improve detection of additional SNPs associated with CAD and BP, the cFDR was computed for each SNP where CAD was the principal trait conditioned on the BP-related SNPs. Ole A. Andreassen *et al*. define the conditional FDR as the posterior probability that a given SNP is null for the first phenotype given that the p-values for both phenotypes are as small or smaller as the observed p-values. cFDR was expressed as:

1 $$cFDR(pi|pj)={\rm{\Pr }}({H}_{0}^{(i)}|Pi\le pi,\,Pj\le pj)$$

To visualize the localization of SNPs associated with CAD given their association with BP, conditional Manhattan plots was constructed to mark the significant SNPs and their chromosomal locations. The 22 chromosomal locations are plotted on the x-axis, and the −log_10_(FDR) CAD values conditional on DBP/SBP are plotted on the y-axis and *vice versa* for BP. As illustrated in Figs S2A and S2B for CAD conditional on DBP/SBP, the small points shown above the red line (−log_10_ cFDR > 1.3, i.e. cFDR < 0.05) represent the SNPs for CAD. A similar procedure was used in the conditional Manhattan plots for BP given CAD (Figs S2C and S2D).

### Conjunction statistics and conjunction Manhattan plots

In order to discover the pleiotropic SNPs associated with both CAD and BP, the conjunctional cFDR (ccFDR) was calculated, which is defined as the posterior probability that a given SNP is null for both phenotypes simultaneously when the *P*-values for both phenotypes are as small or smaller than the observed *P*-values, and given by

2 $${\rm{Conjunction}}\,FD{R}_{i\& j}=\,{\rm{\max }}(cFD{R}_{i|j},\,cFD{R}_{j|i})$$

To visualize the localization of the significant pleiotropic SNPs, ccFDR Manhattan plots were constructed. As illustrated in Fig. 3, the SNPs shown above the red line (ccFDR < 0.05) were SNPs for both CAD and BP.

### Functional term enrichment analysis

Function term enrichment analysis was performed in the gene ontology (GO) terms database (http://geneontology.org/) to describe the biological functions of individual traits related loci^57^. All significant genes were annotated by using three main categories (biological processes, cellular component and molecular functions) to evaluate biological knowledge. This analysis provided comprehensive biological information to partially validate our findings by determining specific genes that are enriched in CAD- and BP-related GO terms.

## Supplementary information

Supplementary Information
