# Supplementary material for: Improved detection of common variants in coronary artery disease and blood pressure using a pleiotropy cFDR method
Source: Sci Rep. 2019 Jul 17;9:10340. doi: 10.1038/s41598-019-46808-2 (PMC6637206; doi:10.1038/s41598-019-46808-2)
Supplement: Supplementary file 1 — Supplementary Information [file 41598_2019_46808_MOESM1_ESM.docx]

**Title:**

Improved detection of common variants in coronary artery disease and blood pressure using a pleiotropy cFDR method

**Authors:**

Xiang-Jie Mao^1^, Qiang Zhang^1^, Fei Xu^1^, Pan Gao^1^, Nan Sun^2^, Bo Wang^1^, Qi-Xin Tang^1^, Yi-bin Hao^3^, Chang-Qing Sun^1*^

**Authors affiliations:**

^1^College of Public Health, Zhengzhou University, 100 Kexue Avenue, Zhengzhou, 450001, Henan, People's Republic of China.

^2^Department of Management Information Systems, Terry College of Business, University of Georgia, Athens, Georgia, United State.

^3^People's Hospital of Zhengzhou, Zhengzhou, 450000, Henan, People's Republic of China.

^*^ **Corresponding author**

Prof. Chang-qing Sun

Department of Social Medicine and Health Management,

College of Public Health, Zhengzhou University

100 Kexue Avenue, Zhengzhou, 450001, Henan, PR China

Phone: +86 371 67781926

Fax: +86 371 67781919

E-mail: [zzugwsy@163.com](mailto:zzugwsy@163.com)

**Supplementary Information**

**Table S1.** Overlapped individuals of datasets between BP and CAD.

**Table S2.** Conditional FDR value of discovery analysis for CAD loci given DBP (cFDR < 0.05).

**Table S3.** Conditional FDR value of discovery analysis for CAD loci given SBP (cFDR < 0.05).

**Table S4.** Conditional FDR value of discovery analysis for CAD loci (cFDR < 0.05).

**Table S5.** SNPs in high LD (R^2^ > 0.6) with CAD-associated loci.

**Table S6.** Conditional FDR value of discovery analysis for DBP loci given CAD (cFDR < 0.05).

**Table S7.** Conditional FDR value of discovery analysis for SBP loci given CAD (cFDR < 0.05).

**Table S8.** Conditional FDR value of discovery analysis for BP loci (cFDR < 0.05).

**Table S9.** SNPs in high LD (R^2^ > 0.6) with BP-associated loci.

**Table S10.** Conjunctional cFDR: pleiotropic loci of discovery analysis in CAD and DBP (ccFDR < 0.05).

**Table S11.** Conjunctional cFDR: pleiotropic loci of discovery analysis in CAD and SBP (ccFDR < 0.05).

**Table S12.** Conditional FDR value for CAD loci of replication analysis (cFDR < 0.05).

**Table S13.** Conditional FDR value for BP loci of replication analysis (cFDR < 0.05).

**Table S14.** Conjunctional cFDR: pleiotropic loci in CAD and BP of replication analysis (ccFDR < 0.05).

**Figure S1.** Stratified True Discovery Rate (TDR) plots for CAD and BP.

**Figure S2.** Figure S2 “Conditional Manhattan Plot” of -log_10_ (cFDR) values for CAD and BP in discovery analysis.

**Figure S3.** Stratified Q-Q plots of replication analysis for CAD and BP.

**Table S1. Overlapped individuals of datasets between BP and CAD**

| **ICBP** | **case** | **control** | **CARDIoGRAM** | **case** | **control** | **C4D** | **case** | **control** |
| --- | --- | --- | --- | --- | --- | --- | --- | --- |
| European studies |  |  | European studies |  |  | European studies |  |  |
| AGES Reykjavik | 3219 |  | ADVANCE | 278 | 312 | **PROCARDIS^b^** | 5720 | 4381 |
| Amish | 1598 |  | CADomics | 2078 | 2952 | HPS (GWAS) | 2704 | 2887 |
| ARIC | 8052 |  | CHARGE | 2287 | 22024 | South Asian studies |  |  |
| B58C–T1DGC | 2580 |  | deCODE CAD | 6640 | 27611 | PROMIS | 4255 | 4098 |
| B58C–WTCCC | 1473 |  | GERMIFS I | 884 | 1604 | LOLIPOP | 2741 | 3696 |
| BLSA | 708 |  | GERMIFS II | 1222 | 1287 |  |  |  |
| CHS | 3277 |  | GERMIFS III (KORA) | 1157 | 1748 |  |  |  |
| CoLaus | 4969 |  | LURIC/AtheroRemo 1 | 652 | 213 |  |  |  |
| EPIC- Norfolk | 2100 |  | LURIC/AtheroRemo 2 | 486 | 296 |  |  |  |
| ERF (EUROSPAN) | 1240 |  | MedStar | 874 | 447 |  |  |  |
| Fenland | 1401 |  | **MIGen^a^** | 1274 | 1407 |  |  |  |
| FHS | 8096 |  | OHGS1 | 1542 | 1455 |  |  |  |
| InCHIANTI | 562 |  | PennCATH | 933 | 468 |  |  |  |
| KORA | 1644 |  | WTCCC | 1926 | 2938 |  |  |  |
| MICROS (EUROSPAN) | 1096 |  |  |  |  |  |  |  |
| NFBC1966 | 4761 |  |  |  |  |  |  |  |
| NSPHS (EUROSPAN) | 644 |  |  |  |  |  |  |  |
| ORCADES (EUROSPAN) | 700 |  |  |  |  |  |  |  |
| RS-I | 4737 |  |  |  |  |  |  |  |
| RS-II | 1760 |  |  |  |  |  |  |  |
| SardiNIA | 3998 |  |  |  |  |  |  |  |
| SHIP | 3306 |  |  |  |  |  |  |  |
| SU.VI.MAX | 1673 |  |  |  |  |  |  |  |
| TwinsUK | 873 |  |  |  |  |  |  |  |
| VIS (EUROSPAN) | 697 |  |  |  |  |  |  |  |
| DGI |  | 1277 |  |  |  |  |  |  |
| FUSION |  | 1038 |  |  |  |  |  |  |
| **MIGen^a^** |  | 1121 |  |  |  |  |  |  |
| **PROCARDIS^b^** |  | 795 |  |  |  |  |  |  |
| Total | 65164 | 4231 |  | 22233 | 64762 |  | 15420 | 15062 |

**Notes:** No overlapped individuals between CARDIoGRAM and C4D.

**^a^**MIGen was overlapped samples between CARDIoGRAM and ICBP.

**^b^**PROCARDIS was overlapped samples between C4D and ICBP.

**Table S2. Conditional FDR value of discovery analysis for CAD loci given DBP** **(cFDR < 0.05)**

| **RSID** | **ROLE** | **GENE** | **CHR** | **BP** | **A1** | **A2** | **P.valueA** | **P.valueB** | **cFDR.AcB** |
| --- | --- | --- | --- | --- | --- | --- | --- | --- | --- |
| rs10965212 | ncRNA_intronic | *CDKN2B-AS1* | chr9 | 22023795 | T | A | 1.37E-17 | 0.0219 | 3.44×10^-14^ |
| rs4510208 | intronic | *ICA1L* | chr2 | 203734866 | C | A | 4.29E-11 | 0.0944 | 2.29×10^-7^ |
| rs11066301 | intronic | *PTPN11* | chr12 | 112871372 | A | G | 5.20E-07 | 6.59E-11 | 1.04×10^-6^ |
| rs10744777 | intronic | *ALDH2* | chr12 | 112233018 | T | C | 1.52E-06 | 6.62E-07 | 4.56×10^-6^ |
| rs10774625 | intronic | *ATXN2* | chr12 | 111910219 | A | G | 7.19E-06 | 4.31E-14 | 7.19×10^-6^ |
| rs2876303 | intronic | *PHACTR1* | chr6 | 12919867 | G | A | 9.10E-09 | 0.0211 | 2.22×10^-5^ |
| rs7970490 | intronic | *CUX2* | chr12 | 111756438 | A | G | 2.18E-05 | 1.48E-07 | 2.91×10^-5^ |
| rs964184 | UTR3 | *ZPR1* | chr11 | 116648917 | G | C | 8.02E-10 | 0.959 | 2.99×10^-5^ |
| rs4767293 | intergenic | *ERP29, NAA25* | chr12 | 112463296 | A | G | 1.81E-05 | 1.52E-06 | 4.07×10^-5^ |
| rs9381462 | intronic | *PHACTR1* | chr6 | 12873775 | A | G | 5.13E-09 | 0.231 | 4.51×10^-5^ |
| rs2347252 | intronic | *MRAS* | chr3 | 138095525 | T | C | 9.83E-08 | 0.0264 | 9.91×10^-5^ |
| rs7651039 | intronic | *BTD* | chr3 | 15648004 | T | C | 1.85E-08 | 0.419 | 1.78×10^-4^ |
| rs1029212 | ncRNA_intronic | *LINC01312, TARID* | chr6 | 134171479 | A | G | 6.23E-08 | 0.723 | 8.72×10^-4^ |
| rs7698460 | intergenic | *GUCY1A3, GUCY1B3* | chr4 | 156666219 | G | A | 0.0010278 | 5.02E-06 | 2.06×10^-3^ |
| rs17514846 | intronic | *FURIN* | chr15 | 91416550 | C | A | 2.37E-05 | 0.00432 | 2.26×10^-3^ |
| rs7902587 | intergenic | *OBFC1, SLK* | chr10 | 105694301 | C | T | 7.07E-05 | 0.00185 | 3.37×10^-3^ |
| rs11066322 | intronic | *PTPN11* | chr12 | 112922529 | G | A | 0.0017581 | 8.15E-06 | 3.52×10^-3^ |
| rs4773144 | intronic | *COL4A2* | chr13 | 110960712 | A | G | 4.15E-07 | 0.691 | 4.76×10^-3^ |
| rs9515203 | intronic | *COL4A2* | chr13 | 111049623 | T | C | 3.42E-05 | 0.0112 | 6.66×10^-3^ |
| rs16824790 | intergenic | *TEX41, PABPC1P2* | chr2 | 146106518 | T | C | 6.97E-05 | 0.00534 | 6.79×10^-3^ |
| rs2252641 | ncRNA_intronic | *TEX41* | chr2 | 145801461 | T | C | 1.37E-05 | 0.0314 | 6.96×10^-3^ |
| rs6489979 | intronic | *CUX2* | chr12 | 111614736 | T | C | 0.0001713 | 0.00203 | 7.44×10^-3^ |
| rs1077393 | intronic | *BAG6* | chr6 | 31610529 | A | G | 0.0005174 | 0.000562 | 9.57×10^-3^ |
| rs6713510 | ncRNA_intronic | *LOC646736* | chr2 | 227034499 | G | A | 9.77E-05 | 0.0104 | 1.37×10^-2^ |
| rs4678408 | intergenic | *NME9, MRAS* | chr3 | 138053187 | A | G | 0.0004686 | 0.001 | 1.37×10^-2^ |
| rs9832013 | intronic | *GPR149* | chr3 | 154118841 | A | G | 2.53E-05 | 0.0709 | 1.85×10^-2^ |
| rs17070864 | intronic | *CSMD1* | chr8 | 4481970 | A | C | 0.0002702 | 0.00632 | 2.12×10^-2^ |
| rs1829867 | intronic | *FNDC3B* | chr3 | 172093296 | G | A | 0.0001055 | 0.0213 | 2.16×10^-2^ |
| rs805293 | intronic | *LY6G6C* | chr6 | 31688518 | T | A | 0.0004996 | 0.00378 | 2.61×10^-2^ |
| rs6474069 | ncRNA_intronic | *LINC00968, LOC101929415* | chr8 | 57437426 | T | C | 9.83E-06 | 0.193 | 2.68×10^-2^ |
| rs2812 | UTR3 | *PECAM1* | chr17 | 62401118 | T | C | 0.0004253 | 0.00378 | 2.76×10^-2^ |
| rs9559759 | intronic | *COL4A1* | chr13 | 110925809 | C | T | 9.72E-06 | 0.239 | 2.95×10^-2^ |
| rs9645499 | ncRNA_intronic | *LOC101928994* | chr10 | 70986610 | G | A | 0.001095 | 0.00162 | 3.04×10^-2^ |
| rs7534861 | intronic | *1-Mar* | chr1 | 220985900 | G | A | 4.77E-05 | 0.0703 | 3.17×10^-2^ |
| rs7069531 | intronic | *CACNB2* | chr10 | 18683267 | A | G | 0.0021776 | 0.000721 | 3.17×10^-2^ |
| rs1868357 | intergenic | *MCTP2, LOC440311* | chr15 | 95375754 | A | T | 5.71E-05 | 0.0724 | 3.35×10^-2^ |
| rs4722680 | intergenic | *EVX1, HIBADH* | chr7 | 27319814 | A | T | 0.0218208 | 5.48E-06 | 4.05×10^-2^ |
| rs2542929 | intergenic | *CPS1, ERBB4* | chr2 | 211711593 | T | G | 0.0002071 | 0.0279 | 4.10×10^-2^ |
| rs11617955 | intronic | *COL4A1* | chr13 | 110818102 | T | A | 1.55E-05 | 0.292 | 4.32×10^-2^ |
| rs2503857 | intergenic | *RASGEF1A, FXYD4* | chr10 | 43769560 | G | A | 0.001705 | 0.00221 | 4.67×10^-2^ |
| rs4962153 | intronic | *ADAMTS13* | chr9 | 136323754 | A | G | 9.01E-05 | 0.0653 | 4.75×10^-2^ |
| rs3818717 | exonic | *RAI1* | chr17 | 17707105 | T | C | 5.16E-06 | 0.98 | 4.92×10^-2^ |

**Note:** All loci with SNPs with cFDR < 0.05 were used to define the number of the loci. The findings of coronary artery disease loci given diastolic blood pressure are presented in the above table.

**RSID:** SNP ID (rs number)**, CHR:** chromosome**, BP:** base pair**, P value A:** p-value of coronary artery disease**, P value B:** p-value of diastolic blood pressure**, cFDR:** conditional false discovery rate**.**

**Table S3. Conditional FDR value of discovery analysis for CAD loci given SBP (cFDR < 0.05)**

| **RSID** | **ROLE** | **GENE** | **CHR** | **BP** | **A1** | **A2** | **P.valueA** | **P.valueB** | **cFDR.AcB** |
| --- | --- | --- | --- | --- | --- | --- | --- | --- | --- |
| rs10965212 | ncRNA_intronic | *CDKN2B-AS1* | chr9 | 22023795 | T | A | 1.37E-17 | 0.22 | 3.39×10^-13^ |
| rs11066301 | intronic | *PTPN11* | chr12 | 112871372 | A | G | 5.20E-07 | 4.94E-08 | 1.04×10^-6^ |
| rs4510208 | intronic | *ICA1L* | chr2 | 203734866 | C | A | 4.29E-11 | 0.946 | 2.37×10^-6^ |
| rs10774625 | intronic | *ATXN2* | chr12 | 111910219 | A | G | 7.19E-06 | 1.13E-09 | 7.19×10^-6^ |
| rs964184 | UTR3 | *ZPR1* | chr11 | 116648917 | G | C | 8.02E-10 | 0.245 | 1.11×10^-5^ |
| rs10744777 | intronic | *ALDH2* | chr12 | 112233018 | T | C | 1.52E-06 | 6.24E-06 | 1.29×10^-5^ |
| rs2876303 | intronic | *PHACTR1* | chr6 | 12919867 | G | A | 9.10E-09 | 0.0169 | 1.81×10^-5^ |
| rs4767293 | intergenic | *ERP29, NAA25* | chr12 | 112463296 | A | G | 1.81E-05 | 7.98E-06 | 8.15×10^-5^ |
| rs9381462 | intronic | *PHACTR1* | chr6 | 12873775 | A | G | 5.13E-09 | 0.153 | 8.86×10^-5^ |
| rs17514846 | intronic | *FURIN* | chr15 | 91416550 | C | A | 2.37E-05 | 1.17E-05 | 9.01×10^-5^ |
| rs7651039 | intronic | *BTD* | chr3 | 15648004 | T | C | 1.85E-08 | 0.254 | 1.07×10^-4^ |
| rs7970490 | intronic | *CUX2* | chr12 | 111756438 | A | G | 2.18E-05 | 2.83E-05 | 1.13×10^-4^ |
| rs2347252 | intronic | *MRAS* | chr3 | 138095525 | T | C | 9.83E-08 | 0.0391 | 2.21×10^-4^ |
| rs1029212 | ncRNA_intronic | *LINC01312, TARID* | chr6 | 134171479 | A | G | 6.23E-08 | 0.673 | 8.08×10^-4^ |
| rs4243111 | intergenic | *BCAR1, CFDP1* | chr16 | 75320827 | C | T | 9.27E-05 | 0.000226 | 8.74×10^-4^ |
| rs9515203 | intronic | *COL4A2* | chr13 | 111049623 | T | C | 3.42E-05 | 0.00145 | 1.26×10^-3^ |
| rs1077393 | intronic | *BAG6* | chr6 | 31610529 | A | G | 0.0005174 | 2.22E-06 | 1.38×10^-3^ |
| rs2812 | UTR3 | *PECAM1* | chr17 | 62401118 | T | C | 0.0004253 | 7.43E-05 | 2.31×10^-3^ |
| rs805293 | intronic | *LY6G6C* | chr6 | 31688518 | T | A | 0.0004996 | 0.000109 | 2.75×10^-3^ |
| rs7902587 | intergenic | *OBFC1, SLK* | chr10 | 105694301 | C | T | 7.07E-05 | 0.00225 | 3.00×10^-3^ |
| rs4773144 | intronic | *COL4A2* | chr13 | 110960712 | A | G | 4.15E-07 | 0.523 | 3.58×10^-3^ |
| rs2252641 | ncRNA_intronic | *TEX41* | chr2 | 145801461 | T | C | 1.37E-05 | 0.019 | 6.11×10^-3^ |
| rs6713510 | ncRNA_intronic | *LOC646736* | chr2 | 227034499 | G | A | 9.77E-05 | 0.00544 | 7.03×10^-3^ |
| rs7698460 | intergenic | *GUCY1A3, GUCY1B3* | chr4 | 156666219 | G | A | 0.0010278 | 0.000509 | 1.07×10^-2^ |
| rs11066322 | intronic | *PTPN11* | chr12 | 112922529 | G | A | 0.0017581 | 0.000225 | 1.13×10^-2^ |
| rs16824790 | intergenic | *TEX41, PABPC1P2* | chr2 | 146106518 | T | C | 6.97E-05 | 0.018 | 1.64×10^-2^ |
| rs7534861 | intronic | *1-Mar* | chr1 | 220985900 | G | A | 4.77E-05 | 0.0301 | 1.66×10^-2^ |
| rs4539564 | intergenic | *ADAMTS7, MORF4L1* | chr15 | 79128499 | G | A | 9.46E-06 | 0.108 | 1.92×10^-2^ |
| rs4678408 | intergenic | *NME9, MRAS* | chr3 | 138053187 | A | G | 0.0004686 | 0.00391 | 2.26×10^-2^ |
| rs11067009 | intergenic | *RBM19, TBX5* | chr12 | 114673596 | G | T | 8.66E-06 | 0.264 | 2.59×10^-2^ |
| rs6489979 | intronic | *CUX2* | chr12 | 111614736 | T | C | 0.0001713 | 0.0143 | 2.65×10^-2^ |
| rs6068963 | intergenic | *DOK5, LINC01441* | chr20 | 53325604 | G | A | 9.55E-06 | 0.224 | 2.68×10^-2^ |
| rs4290163 | intergenic | *CYP17A1, BORCS7* | chr10 | 104610926 | G | T | 9.00E-05 | 0.0371 | 2.95×10^-2^ |
| rs7643852 | intergenic | *ZNF621, CTNNB1* | chr3 | 41110710 | A | G | 0.0002582 | 0.0109 | 3.14×10^-2^ |
| rs3818717 | exonic | *RAI1* | chr17 | 17707105 | T | C | 5.16E-06 | 0.511 | 3.38×10^-2^ |
| rs1011970 | ncRNA_intronic | *CDKN2B-AS1* | chr9 | 22062134 | G | T | 6.37E-06 | 0.438 | 3.55×10^-2^ |
| rs13154066 | intergenic | *NPR3, LOC340113* | chr5 | 32831670 | T | C | 0.0269392 | 2.12E-07 | 3.59×10^-2^ |
| rs4415546 | intergenic | *ZNF326, BARHL2* | chr1 | 90658051 | G | T | 5.56E-06 | 0.695 | 3.73×10^-2^ |
| rs998584 | intergenic | *VEGFA, LINC01512* | chr6 | 43757896 | C | A | 0.009024 | 9.30E-05 | 4.11×10^-2^ |
| rs13070927 | intronic | *FGD5* | chr3 | 14919646 | G | T | 0.0099472 | 0.000113 | 4.16×10^-2^ |
| rs366590 | intronic | *PLEKHA7* | chr11 | 16872440 | G | A | 0.0160067 | 3.78E-06 | 4.40×10^-2^ |
| rs2542929 | intergenic | *CPS1, ERBB4* | chr2 | 211711593 | T | G | 0.0002071 | 0.028 | 4.45×10^-2^ |
| rs10195020 | intergenic | *CPS1, ERBB4* | chr2 | 211657226 | T | G | 8.88E-05 | 0.0629 | 4.47×10^-2^ |
| rs9832013 | intronic | *GPR149* | chr3 | 154118841 | A | G | 2.53E-05 | 0.248 | 4.75×10^-2^ |

**Note:** All loci with SNPs with cFDR < 0.05 were used to define the number of the loci. The findings of coronary artery disease loci given systolic blood pressure are presented in the above table.

**RSID:** SNP ID (rs number), **CHR:** chromosome, **BP:** base pair, **P value A:** p-value of coronary artery disease, **P value B:** p-value of systolic blood pressure, **cFDR:** conditional false discovery rate.

**Table S4. Conditional FDR value of discovery analysis for CAD loci (cFDR < 0.05)**

| **CHR** | **RSID** | **Gene** | **Role** | **Discovery analysis** | |  | **Replication analysis** | | **SNP type^b^** |
| --- | --- | --- | --- | --- | --- | --- | --- | --- | --- |
|  |  |  |  | **P.CAD** | **cFDR** |  | **SNP** | **cFDR** |  |
| chr1 | rs7534861 | *1-Mar(MARC1)* | intronic | 4.77E-05 | 1.66E-02 |  |  |  | Novel |
| chr1 | rs4415546 | *ZNF326,BARHL2* | intergenic | 5.56E-06 | 3.73E-02 |  |  |  | Novel |
| chr10 | rs7902587 | *OBFC1,* ***SLK*^a^** | intergenic | 7.07E-05 | 3.00E-03 |  |  |  | Novel |
| chr10 | rs4290163 | ***CYP17A1*^a^*,BORCS7*^a^** | intergenic | 9.00E-05 | 2.95E-02 |  |  |  | Novel |
| chr10 | rs9645499 | *LOC101928994* | ncRNA_intronic | 1.10E-03 | 3.04E-02 |  |  |  | Novel |
| chr10 | rs7069531 | *CACNB2* | intronic | 2.18E-03 | 3.17E-02 |  |  |  | Novel |
| chr10 | rs2503857 | *RASGEF1A,FXYD4* | intergenic | 1.71E-03 | 4.67E-02 |  |  |  | Novel |
| chr11 | **rs964184** | ***ZPR1*^a^** | UTR3 | 8.02E-10 | 1.11E-05 |  |  |  | **Confirmed** |
| chr11 | rs366590 | ***PLEKHA7*^a^** | intronic | 1.60E-02 | 4.40E-02 |  |  |  | Novel |
| chr12 | **rs11066301** | *PTPN11* | intronic | 5.20E-07 | 1.04E-06 |  |  |  | **LD(CAD)** |
| chr12 | **rs10774625** | ***ATXN2*^a^***** | intronic | 7.19E-06 | 7.19E-06 |  | rs653178,  R2=0.9108 | 4.04E-04 | **Confirmed** |
| chr12 | **rs10744777** | ***ALDH2*^a^** | intronic | 1.52E-06 | 1.29E-05 |  |  |  | **Confirmed** |
| chr12 | **rs4767293** | *ERP29*, NAA25** | intergenic | 1.81E-05 | 8.15E-05 |  | rs4767293 | 2.71E-02 | **LD(CAD)** |
| chr12 | **rs7970490** | ***CUX2*^a^***** | intronic | 2.18E-05 | 1.13E-04 |  | rs7970490 | 1.15E-03 | **LD(CAD)** |
| chr12 | **rs11066322** | *PTPN11* | intronic | 1.76E-03 | 1.13E-02 |  |  |  | **LD(CAD)** |
| chr12 | rs11067009 | *RBM19,TBX5* | intergenic | 8.66E-06 | 2.59E-02 |  |  |  | Novel |
| chr12 | **rs6489979** | ***CUX2*^a^***** | intronic | 1.71E-04 | 2.65E-02 |  |  |  | **LD(CAD)** |
| chr13 | **rs9515203** | ***COL4A2*^a^** | intronic | 3.42E-05 | 1.26E-03 |  |  |  | **Confirmed** |
| chr13 | **rs4773144** | ***COL4A2*^a^** | intronic | 4.15E-07 | 3.58E-03 |  |  |  | **Confirmed** |
| chr13 | **rs9559759** | ***COL4A1*^a^** | intronic | 9.72E-06 | 2.95E-02 |  |  |  | **LD(CAD)** |
| chr13 | **rs11617955** | ***COL4A1*^a^** | intronic | 1.55E-05 | 4.32E-02 |  |  |  | **Confirmed** |
| chr15 | **rs17514846** | ***FURIN*^a^***** | intronic | 2.37E-05 | 9.01E-05 |  | rs17514846 | 2.44E-03 | **Confirmed** |
| chr15 | **rs4539564** | ***ADAMTS7*^a^*,MORF4L1*^a^** | intergenic | 9.46E-06 | 1.92E-02 |  |  |  | **LD(CAD)** |
| chr15 | rs1868357 | ***MCTP2*^a^*,****LOC440311* | intergenic | 5.71E-05 | 3.35E-02 |  |  |  | Novel |
| chr16 | **rs4243111** | *BCAR1,****CFDP1*^a^** | intergenic | 9.27E-05 | 8.74E-04 |  |  |  | **LD(CAD)** |
| chr17 | **rs2812** | ***PECAM1* ^a^** | UTR3 | 4.25E-04 | 2.31E-03 |  |  |  | **LD(CAD)** |
| chr17 | **rs3818717** | ***RAI1*^a^** | exonic | 5.16E-06 | 3.38E-02 |  |  |  | **LD(CAD)** |
| chr2 | **rs4510208** | *ICA1L* | intronic | 4.29E-11 | 2.37E-06 |  |  |  | **LD(CAD)** |
| chr2 | **rs2252641** | ***TEX41*^a^** | ncRNA_intronic | 1.37E-05 | 6.11E-03 |  |  |  | **Confirmed** |
| chr2 | rs6713510 | *LOC646736* | ncRNA_intronic | 9.77E-05 | 7.03E-03 |  |  |  | Novel |
| chr2 | rs16824790 | ***TEX41*^a^***,PABPC1P2* | intergenic | 6.97E-05 | 1.64E-02 |  |  |  | Novel |
| chr2 | rs2542929 | *CPS1,ERBB4* | intergenic | 2.07E-04 | 4.45E-02 |  |  |  | Novel |
| chr2 | rs10195020 | *CPS1,ERBB4* | intergenic | 8.88E-05 | 4.47E-02 |  |  |  | Novel |
| chr20 | rs6068963 | *DOK5,LINC01441* | intergenic | 9.55E-06 | 2.68E-02 |  |  |  | Novel |
| chr3 | **rs7651039** | ***BTD*^a^** | intronic | 1.85E-08 | 1.07E-04 |  |  |  | **Confirmed** |
| chr3 | **rs2347252** | ***MRAS*^a^** | intronic | 9.83E-08 | 2.21E-04 |  |  |  | **LD(CAD)** |
| chr3 | rs4678408 | *NME9,****MRAS*^a^** | intergenic | 4.69E-04 | 2.26E-02 |  |  |  | Novel |
| chr3 | rs7643852 | *ZNF621,CTNNB1* | intergenic | 2.58E-04 | 3.14E-02 |  |  |  | Novel |
| chr3 | **rs13070927** | ***FGD5*^a^** | intronic | 9.95E-03 | 4.16E-02 |  |  |  | **LD(CAD)** |
| chr3 | **rs9832013** | ***GPR149*^a^** | intronic | 2.53E-05 | 4.75E-02 |  |  |  | **LD(CAD)** |
| chr3 | rs1829867 | ***FNDC3B*^a^** | intronic | 1.06E-04 | 2.16E-02 |  |  |  | Novel |
| chr4 | **rs7698460** | *GUCY1A3*,GUCY1B3** | intergenic | 1.03E-03 | 1.07E-02 |  |  |  | **LD(CAD)** |
| chr5 | rs13154066 | *NPR3,LOC340113* | intergenic | 2.69E-02 | 3.59E-02 |  |  |  | Novel |
| chr6 | **rs2876303** | ***PHACTR1*^a^***** | intronic | 9.10E-09 | 1.81E-05 |  | rs2876303 | 2.06E-07 | **LD(CAD)** |
| chr6 | **rs9381462** | ***PHACTR1*^a^***** | intronic | 5.13E-09 | 8.86E-05 |  | rs9381462 | 1.65E-03 | **Confirmed** |
| chr6 | **rs1029212** | ***LINC01312*^a^*,TARID*^a^** | ncRNA_intronic | 6.23E-08 | 8.08E-04 |  |  |  | **LD(CAD)** |
| chr6 | **rs1077393** | *BAG6* | intronic | 5.17E-04 | 1.38E-03 |  |  |  | **LD(CAD)** |
| chr6 | rs805293 | *LY6G6C* | intronic | 5.00E-04 | 2.75E-03 |  |  |  | Novel |
| chr6 | rs998584 | ***VEGFA*^a^***,LINC01512* | intergenic | 9.02E-03 | 4.11E-02 |  |  |  | Novel |
| chr7 | rs4722680 | *EVX1,HIBADH* | intergenic | 2.18E-02 | 4.05E-02 |  |  |  | Novel |
| chr8 | rs17070864 | *CSMD1* | intronic | 2.70E-04 | 2.12E-02 |  |  |  | Novel |
| chr8 | rs6474069 | *LINC00968,LOC101929415* | ncRNA_intronic | 9.83E-06 | 2.68E-02 |  |  |  | Novel |
| chr9 | **rs10965212** | ***CDKN2B-AS1*^a^***** | ncRNA_intronic | 1.37E-17 | 3.39E-13 |  | rs7049105,  R2=0.94568 | 1.79E-10 | **LD(CAD)** |
| chr9 | **rs1011970** | ***CDKN2B-AS1*^a^***** | ncRNA_intronic | 6.37E-06 | 3.55E-02 |  |  |  | **LD(CAD)** |
| chr9 | rs4962153 | *ADAMTS13* | intronic | 9.01E-05 | 4.75E-02 |  |  |  | Novel |

**Notes:** All loci with SNPs with cFDR < 0.05 were used to define the number of the loci. The R^2^ is the measure of linkage disequilibrium (LD) between the identified SNP and the SNP which is significant in the replication analysis or other CAD studies. If the R^2^ value is greater than 0.6, it represents that these two SNPs are in high LD, this SNP considered to be replicated/reported.

**^a^**Genes identified in our study have been reported to be associated with CAD in original CAD GWAS and previous GWAS studies.

**^b^SNP type** means whether SNPs identified in our study compared to the original CAD GWAS and previous studies are Novel or Confirmed or in high LD with CAD-associated loci.

**^*^**Genes identified in discovery analysis further confirmed to be associated with CAD in the replication analysis.

**Table S5.** SNPs in high LD (R^2^ > 0.6) with CAD-associated loci.

| **SNP** | **R^2^** | **Proxy SNP** | **Trait** |
| --- | --- | --- | --- |
| rs11066301 | 0.9879 | rs11066320 | CAD |
| rs4767293 | 0.89342 | rs2238151 | CAD |
| rs7970490 | 0.75237 | rs7978923 | CAD |
| rs11066322 | 0.95641 | rs11066284 | CAD |
| rs6489979 | 0.60058 | rs7398796 | CAD |
| rs9559759 | 0.92331 | rs11619038 | CAD |
| rs4539564 | 0.79977 | rs7173743 | CAD |
| rs4243111 | 0.97618 | rs4888378 | CAD |
| rs2812 | 0.99541 | rs9892152 | CAD |
| rs3818717 | 0.69389 | rs1889014 | CAD |
| rs4510208 | 1 | rs6725887 | CAD |
| rs2347252 | 1 | rs2306374 | CAD |
| rs13070927 | 0.94897 | rs13079221 | CAD |
| rs9832013 | 0.84979 | rs115162635 | CAD |
| rs7698460 | 0.97612 | rs11721947 | CAD |
| rs2876303 | 0.82383 | rs12526453 | CAD |
| rs1029212 | 0.9422 | rs12202017 | CAD |
| rs1077393 | 0.79484 | rs2736172 | CAD |
| rs10965212 | 0.98593 | rs7049105 | CAD |
| rs1011970 | 0.96847 | rs28557075 | CAD |

**Table S6. Conditional FDR value of discovery analysis for DBP loci given CAD (cFDR < 0.05)**

| **RSID** | **ROLE** | **GENE** | **CHR** | **BP** | **A1** | **A2** | **P.valueA** | **P.valueB** | **cFDR.BcA** |
| --- | --- | --- | --- | --- | --- | --- | --- | --- | --- |
| rs10774625 | intronic | *ATXN2* | chr12 | 111910219 | A | G | 7.19E-06 | 4.31E-14 | 6.47×10^-13^ |
| rs11066301 | intronic | *PTPN11* | chr12 | 112871372 | A | G | 5.20E-07 | 6.59E-11 | 6.59×10^-10^ |
| rs7970490 | intronic | *CUX2* | chr12 | 111756438 | A | G | 2.18E-05 | 1.48E-07 | 1.38×10^-6^ |
| rs10744777 | intronic | *ALDH2* | chr12 | 112233018 | T | C | 1.52E-06 | 6.62E-07 | 3.64×10^-6^ |
| rs4767293 | intergenic | *ERP29, NAA25* | chr12 | 112463296 | A | G | 1.81E-05 | 1.52E-06 | 1.03×10^-5^ |
| rs2969070 | intergenic | *LOC101927181, GRIFIN* | chr7 | 2512545 | G | A | 0.4292307 | 2.57E-09 | 4.42×10^-5^ |
| rs7698460 | intergenic | *GUCY1A3, GUCY1B3* | chr4 | 156666219 | G | A | 0.0010278 | 5.02E-06 | 2.28×10^-4^ |
| rs11066322 | intronic | *PTPN11* | chr12 | 112922529 | G | A | 0.0017581 | 8.15E-06 | 4.72×10^-4^ |
| rs2588917 | intronic | *C10orf107* | chr10 | 63524979 | C | A | 0.2277748 | 2.43E-07 | 1.70×10^-3^ |
| rs4722680 | intergenic | *EVX1, HIBADH* | chr7 | 27319814 | A | T | 0.0218208 | 5.48E-06 | 2.51×10^-3^ |
| rs1887320 | intergenic | *LOC101929413, LOC339593* | chr20 | 10965998 | G | A | 0.1615325 | 9.94E-07 | 4.04×10^-3^ |
| rs4245909 | intronic | *MECOM* | chr3 | 169172788 | A | G | 0.2073845 | 1.22E-06 | 5.22×10^-3^ |
| rs2643825 | intergenic | *SLC4A7, EOMES* | chr3 | 27562613 | A | G | 0.0365731 | 1.20E-05 | 6.78×10^-3^ |
| rs4952971 | intergenic | *LOC102723854, ZFP36L2* | chr2 | 43373119 | A | G | 0.0172229 | 3.86E-05 | 1.27×10^-2^ |
| rs7902587 | intergenic | *OBFC1, SLK* | chr10 | 105694301 | C | T | 7.07E-05 | 0.00185 | 1.39×10^-2^ |
| rs1077393 | intronic | *BAG6* | chr6 | 31610529 | A | G | 0.0005174 | 0.000562 | 1.69×10^-2^ |
| rs17078838 | intergenic | *RFX6, VGLL2* | chr6 | 117457307 | A | G | 0.4894015 | 3.02E-06 | 1.76×10^-2^ |
| rs17514846 | intronic | *FURIN* | chr15 | 91416550 | C | A | 2.37E-05 | 0.00432 | 2.09×10^-2^ |
| rs10965212 | ncRNA_intronic | *CDKN2B-AS1* | chr9 | 22023795 | T | A | 1.37E-17 | 0.0219 | 2.19×10^-2^ |
| rs6489979 | intronic | *CUX2* | chr12 | 111614736 | T | C | 0.0001713 | 0.00203 | 2.32×10^-2^ |
| rs10865186 | intergenic | *HAAO, LOC102723854* | chr2 | 43193347 | T | C | 0.7423175 | 2.70E-06 | 2.35×10^-2^ |
| rs4678408 | intergenic | *NME9, MRAS* | chr3 | 138053187 | A | G | 0.0004686 | 0.001 | 2.77×10^-2^ |
| rs16855893 | intronic | *NOSTRIN* | chr2 | 169652647 | T | C | 0.0161913 | 8.88E-05 | 2.77×10^-2^ |
| rs998584 | intergenic | *VEGFA, LINC01512* | chr6 | 43757896 | C | A | 0.009024 | 0.000156 | 2.95×10^-2^ |
| rs16824790 | intergenic | *TEX41, PABPC1P2* | chr2 | 146106518 | T | C | 6.97E-05 | 0.00534 | 3.36×10^-2^ |
| rs11812165 | intronic | *C10orf11* | chr10 | 78204760 | G | A | 0.024693 | 0.000113 | 3.67×10^-2^ |
| rs7069531 | intronic | *CACNB2* | chr10 | 18683267 | A | G | 0.0021776 | 0.000721 | 3.76×10^-2^ |
| rs1896334 | intergenic | *TBX3, MED13L* | chr12 | 115415141 | G | A | 0.256346 | 1.68E-05 | 4.07×10^-2^ |

**Note:** All loci with SNPs with cFDR < 0.05 were used to define the number of the loci. The findings of DBP loci given CAD are presented in the above table**.**

**RSID:** SNP ID (rs number)**, CHR:** chromosome, **BP:** base pair, **P value A:** p value of coronary artery disease, **P value B:** p value of diastolic blood pressure, **cFDR:** conditional false discovery rate.

**Table S7. Conditional FDR value of discovery analysis for SBP loci given CAD (cFDR < 0.05)**

| **RSID** | **ROLE** | **GENE** | **CHR** | **BP** | **A1** | **A2** | **P.valueA** | **P.valueB** | **cFDR.BcA** |
| --- | --- | --- | --- | --- | --- | --- | --- | --- | --- |
| rs10774625 | intronic | *ATXN2* | chr12 | 111910219 | A | G | 7.19E-06 | 1.13E-09 | 1.70×10^-8^ |
| rs11066301 | intronic | *PTPN11* | chr12 | 112871372 | A | G | 5.20E-07 | 4.94E-08 | 4.94×10^-7^ |
| rs10744777 | intronic | *ALDH2* | chr12 | 112233018 | T | C | 1.52E-06 | 6.24E-06 | 3.43×10^-5^ |
| rs4767293 | intergenic | *ERP29, NAA25* | chr12 | 112463296 | A | G | 1.81E-05 | 7.98E-06 | 5.39×10^-5^ |
| rs17514846 | intronic | *FURIN* | chr15 | 91416550 | C | A | 2.37E-05 | 1.17E-05 | 6.79×10^-5^ |
| rs1077393 | intronic | *BAG6* | chr6 | 31610529 | A | G | 0.0005174 | 2.22E-06 | 1.33×10^-4^ |
| rs7970490 | intronic | *CUX2* | chr12 | 111756438 | A | G | 2.18E-05 | 2.83E-05 | 1.58×10^-4^ |
| rs13154066 | intergenic | *NPR3, LOC340113* | chr5 | 32831670 | T | C | 0.0269392 | 2.12E-07 | 2.74×10^-4^ |
| rs2812 | UTR3 | *PECAM1* | chr17 | 62401118 | T | C | 0.0004253 | 7.43E-05 | 1.65×10^-3^ |
| rs4243111 | intergenic | *BCAR1, CFDP1* | chr16 | 75320827 | C | T | 9.27E-05 | 0.000226 | 1.87×10^-3^ |
| rs366590 | intronic | *PLEKHA7* | chr11 | 16872440 | G | A | 0.0160067 | 3.78E-06 | 2.33×10^-3^ |
| rs805293 | intronic | *LY6G6C* | chr6 | 31688518 | T | A | 0.0004996 | 0.000109 | 2.36×10^-3^ |
| rs10786156 | intronic | *PLCE1* | chr10 | 96014622 | C | G | 0.0216333 | 3.79E-06 | 2.41×10^-3^ |
| rs2969070 | intergenic | *LOC101927181, GRIFIN* | chr7 | 2512545 | G | A | 0.4292307 | 4.30E-07 | 5.54×10^-3^ |
| rs5068 | ncRNA_intronic | *NPPA-AS1* | chr1 | 11905974 | A | G | 0.7889353 | 1.86E-07 | 5.73×10^-3^ |
| rs9515203 | intronic | *COL4A2* | chr13 | 111049623 | T | C | 3.42E-05 | 0.00145 | 6.63×10^-3^ |
| rs2920151 | intronic | *SBF2* | chr11 | 10296667 | C | T | 0.0674129 | 6.11E-06 | 7.83×10^-3^ |
| rs11066322 | intronic | *PTPN11* | chr12 | 112922529 | G | A | 0.0017581 | 0.000225 | 9.11×10^-3^ |
| rs4667739 | intergenic | *FIGN, GRB14* | chr2 | 165115638 | C | A | 0.3389044 | 1.07E-06 | 1.10×10^-2^ |
| rs7698460 | intergenic | *GUCY1A3, GUCY1B3* | chr4 | 156666219 | G | A | 0.0010278 | 0.000509 | 1.26×10^-2^ |
| rs7902587 | intergenic | *OBFC1, SLK* | chr10 | 105694301 | C | T | 7.07E-05 | 0.00225 | 1.27×10^-2^ |
| rs4245909 | intronic | *MECOM* | chr3 | 169172788 | A | G | 0.2073845 | 2.71E-06 | 1.39×10^-2^ |
| rs853964 | intergenic | *MIR588, RSPO3* | chr6 | 127029267 | G | A | 0.2028131 | 3.96E-06 | 1.42×10^-2^ |
| rs998584 | intergenic | *VEGFA, LINC01512* | chr6 | 43757896 | C | A | 0.009024 | 9.30E-05 | 1.56×10^-2^ |
| rs13070927 | intronic | *FGD5* | chr3 | 14919646 | G | T | 0.0099472 | 0.000113 | 1.68×10^-2^ |
| rs10786152 | intronic | *PLCE1* | chr10 | 95893514 | A | G | 0.8396592 | 1.53E-06 | 2.15×10^-2^ |
| rs4722680 | intergenic | *EVX1, HIBADH* | chr7 | 27319814 | A | T | 0.0218208 | 6.77E-05 | 2.17×10^-2^ |
| rs7787898 | intergenic | *CCDC71L, PIK3CG* | chr7 | 106409897 | T | G | 0.4850123 | 3.91E-06 | 2.26×10^-2^ |
| rs9467445 | intergenic | *CMAHP, LOC101928663* | chr6 | 25234884 | A | G | 0.5136235 | 3.33E-06 | 2.54×10^-2^ |
| rs11070252 | intergenic | *TJP1, GOLGA8J* | chr15 | 30329208 | T | C | 0.4753878 | 5.74E-06 | 2.96×10^-2^ |
| rs6713510 | ncRNA_intronic | *LOC646736* | chr2 | 227034499 | G | A | 9.77E-05 | 0.00544 | 3.32×10^-2^ |
| rs12656502 | intergenic | *FBXL17, LINC01023* | chr5 | 107981747 | G | A | 0.0103173 | 0.000378 | 4.57×10^-2^ |
| rs4812537 | intergenic | *CHD6, PTPRT* | chr20 | 40261579 | T | C | 0.2644259 | 1.90E-05 | 4.74×10^-2^ |

**Note:** All loci with SNPs with cFDR < 0.05 were used to define the number of the loci. The findings of SBP loci given CAD are presented in the above table.

**RSID:** SNP ID (rs number), **CHR:** chromosome, **BP:** base pair, **P value A:** p value of coronary artery disease, **P value B:** p value of systolic blood pressure, **cFDR:** conditional false discovery rate.

**Table S8. Conditional FDR value of discovery analysis for BP loci (cFDR < 0.05)**

| **CHR** | **RSID** | **Gene** | **Role** | **Discovery analysis** | |  | **Replication analysis** | | **SNP type** |
| --- | --- | --- | --- | --- | --- | --- | --- | --- | --- |
|  |  |  |  | **P.BP** | **cFDR** |  | **SNP** | **cFDR** |  |
| chr1 | **rs5068** | NPPA-AS1 | ncRNA_intronic | 1.86E-07 | 5.73E-03 |  |  |  | **Confirmed** |
| chr10 | **rs10786156** | ***PLCE1*^a^***** | intronic | 3.79E-06 | 2.41E-03 |  |  |  | **Confirmed** |
| chr10 | rs7902587 | OBFC1,***SLK*^a^** | intergenic | 2.25E-03 | 1.27E-02 |  |  |  | Novel |
| chr10 | **rs10786152** | ***PLCE1*^a^***** | intronic | 1.53E-06 | 2.15E-02 |  | rs10786152 | 1.10E-02 | **LD(SBP)** |
| chr10 | **rs2588917** | C10orf107* | intronic | 2.43E-07 | 1.70E-03 |  | rs2588917 | 5.62E-03 | **LD(DBP)** |
| chr10 | rs11812165 | C10orf11 | intronic | 1.13E-04 | 3.67E-02 |  |  |  | Novel |
| chr10 | **rs7069531** | ***CACNB2*^a^** | intronic | 7.21E-04 | 3.76E-02 |  |  |  | **LD(BP)** |
| chr11 | **rs366590** | ***PLEKHA7*^a^** | intronic | 3.78E-06 | 2.33E-03 |  |  |  | **LD(BP)** |
| chr11 | **rs2920151** | ***SBF2*^a^** | intronic | 6.11E-06 | 7.83E-03 |  |  |  | **Confirmed** |
| chr12 | **rs10774625** | ***ATXN2*^a^***** | intronic | 1.13E-09 | 1.70E-08 |  | rs653178,  R2=0.9108 | 1.20E-12 | **LD(BP)** |
| chr12 | **rs11066301** | ***PTPN11*^a^** | intronic | 4.94E-08 | 4.94E-07 |  |  |  | **LD(DBP)** |
| chr12 | **rs10744777** | ALDH2 | intronic | 6.24E-06 | 3.43E-05 |  |  |  | **LD(DBP)** |
| chr12 | **rs4767293** | ERP29*,***NAA25*^a^***** | intergenic | 7.98E-06 | 5.39E-05 |  | rs4767293 | 6.20E-04 | **LD(DBP)** |
| chr12 | **rs7970490** | **CUX2^a^*** | intronic | 2.83E-05 | 1.58E-04 |  | rs7970490 | 9.03E-06 | **LD(DBP)** |
| chr12 | **rs11066322** | ***PTPN11*^a^** | intronic | 2.25E-04 | 9.11E-03 |  |  |  | **LD(SBP)** |
| chr12 | **rs6489979** | **CUX2^a^*** | intronic | 2.03E-03 | 2.32E-02 |  |  |  | **Confirmed** |
| chr12 | rs1896334 | TBX3****,MED13L*^a^***** | intergenic | 1.68E-05 | 4.07E-02 |  |  |  | Novel |
| chr13 | rs9515203 | ***COL4A2*^a^** | intronic | 1.45E-03 | 6.63E-03 |  |  |  | Novel |
| chr15 | **rs17514846** | ***FURIN*^a^***** | intronic | 1.17E-05 | 6.79E-05 |  | rs17514846 | 5.27E-04 | **Confirmed** |
| chr15 | rs11070252 | TJP1*,GOLGA8J* | intergenic | 5.74E-06 | 2.96E-02 |  | rs11070252 | 2.73E-02 | Novel |
| chr16 | **rs4243111** | ***BCAR1*^a^*,CFDP1*^a^** | intergenic | 2.26E-04 | 1.87E-03 |  |  |  | **LD(SBP)** |
| chr17 | **rs2812** | *PECAM1* | UTR3 | 7.43E-05 | 1.65E-03 |  |  |  | **LD(SBP)** |
| chr2 | **rs4667739** | FIGN,***GRB14*^a^** | intergenic | 1.07E-06 | 1.10E-02 |  |  |  | **Confirmed** |
| chr2 | rs6713510 | LOC646736 | ncRNA_intronic | 5.44E-03 | 3.32E-02 |  |  |  | Novel |
| chr2 | **rs4952971** | LOC102723854,***ZFP36L2*^a^** | intergenic | 3.86E-05 | 1.27E-02 |  |  |  | **LD(DBP)** |
| chr2 | rs10865186 | HAAO*,LOC102723854 | intergenic | 2.70E-06 | 2.35E-02 |  | rs10865186 | 5.35E-03 | Novel |
| chr2 | rs16855893 | NOSTRIN | intronic | 8.88E-05 | 2.77E-02 |  |  |  | Novel |
| chr2 | rs16824790 | ***TEX41*^a^**,PABPC1P2 | intergenic | 5.34E-03 | 3.36E-02 |  |  |  | Novel |
| chr20 | rs4812537 | ***CHD6*^a^*,***PTPRT | intergenic | 1.90E-05 | 4.74E-02 |  |  |  | Novel |
| chr20 | **rs1887320** | LOC101929413*,  LOC339593 | intergenic | 9.94E-07 | 4.04E-03 |  | rs1887320 | 8.02E-05 | **Confirmed** |
| chr3 | **rs4245909** | ***MECOM*^a^** | intronic | 2.71E-06 | 1.39E-02 |  | rs4245909 | 1.79E-03 | **LD(BP)** |
| chr3 | rs13070927 | ***FGD5*^a^** | intronic | 1.13E-04 | 1.68E-02 |  |  |  | Novel |
| chr3 | **rs2643825** | ***SLC4A7*^a^**,EOMES | intergenic | 1.20E-05 | 6.80E-03 |  |  |  | **LD(DBP)** |
| chr3 | rs4678408 | ***NME9*^a^*,MRAS*^a^** | intergenic | 1.00E-03 | 2.77E-02 |  |  |  | Novel |
| chr4 | **rs7698460** | GUCY1A3*,GUCY1B3* | intergenic | 5.09E-04 | 1.26E-02 |  |  |  | **LD(BP)** |
| chr5 | **rs13154066** | ***NPR3*^a^**,***LOC340113 | intergenic | 2.12E-07 | 2.74E-04 |  | rs13154066 | 1.26E-03 | **Confirmed** |
| chr5 | rs12656502 | FBXL17,LINC01023 | intergenic | 3.78E-04 | 4.57E-02 |  |  |  | Novel |
| chr6 | **rs1077393** | ***BAG6*^a^***** | intronic | 2.22E-06 | 1.33E-04 |  | rs1077393 | 7.85E-03 | **LD(BP)** |
| chr6 | **rs805293** | LY6G6C | intronic | 1.09E-04 | 2.36E-03 |  |  |  | **Confirmed** |
| chr6 | rs853964 | MIR588,***RSPO3*^a^** | intergenic | 3.96E-06 | 1.42E-02 |  |  |  | Novel |
| chr6 | rs998584 | ***VEGFA*^a^*,***LINC01512 | intergenic | 9.30E-05 | 1.56E-02 |  |  |  | Novel |
| chr6 | rs9467445 | CMAHP*,LOC101928663* | intergenic | 3.33E-06 | 2.54E-02 |  | rs9467445 | 2.70E-02 | Novel |
| chr6 | rs17078838 | ***RFX6*^a^**,VGLL2*^a^***** | intergenic | 3.02E-06 | 1.76E-02 |  |  |  | Novel |
| chr7 | **rs2969070** | LOC101927181*,***GRIFIN*^a^***** | intergenic | 4.30E-07 | 5.54E-03 |  | rs2969070 | 9.00E-06 | **Confirmed** |
| chr7 | **rs4722680** | EVX1*,***HIBADH*^a^***** | intergenic | 6.77E-05 | 2.17E-02 |  |  |  | **LD(BP)** |
| chr7 | **rs7787898** | CCDC71L,PIK3CG | intergenic | 3.91E-06 | 2.26E-02 |  |  |  | **Confirmed** |
| chr9 | rs10965212 | ***CDKN2B-AS1*^a^***** | ncRNA_intronic | 2.19E-02 | 2.19E-02 |  | rs7049105,  R2=0.99602 | 4.14E-02 | Novel |

**Notes:** All loci with SNPs with cFDR < 0.05 were used to define the number of the loci. The R^2^ is the measure of LD between the identified SNP and the SNP which is significant in the replication analysis or other BP studies. If the R^2^ value is greater than 0.6, it represents that these two SNPs are in high LD, this SNP considered to be replicated/reported.

**^a^**Genes identified in our study have been reported to be associated with BP in original and previous BP GWAS studies.

**^b^SNP type** means whether SNPs identified in our study compared to the original BP GWAS and previous studies are Novel or Confirmed or in high LD with BP-associated loci.

**^*^**Genes identified in discovery analysis further confirmed to be associated with BP in the replication analysis.

**Table S9.** SNPs in high LD (R^2^ > 0.6) with BP-associated loci.

| **SNP** | **R^2^** | **Proxy SNP** | **Trait** |
| --- | --- | --- | --- |
| rs10774625 | 0.81445 | rs932764 | SBP |
| rs2588917 | 0.77593 | rs2675618 | DBP |
| rs7069531 | 0.81767 | rs11014166 | SBP |
| rs366590 | 0.65411 | rs381815 | SBP/DBP |
| rs10774625 | 0.94946 | rs3184504 | SBP/DBP |
| rs11066301 | 0.92124 | rs17630235 | DBP |
| rs10744777 | 0.99539 | rs2238151 | DBP |
| rs4767293 | 0.89342 | rs2238151 | DBP/SBP |
| rs7970490 | 0.67245 | rs2238151 | DBP/SBP |
| rs11066322 | 0.9868 | rs11066284 | SBP |
| rs4243111 | 0.90903 | rs11643209 | SBP |
| rs2812 | 0.9954 | rs9902260 | SBP |
| rs4952971 | 0.93731 | rs7349377 | DBP |
| rs4245909 | 0.80779 | rs419076 | SBP/DBP |
| rs2643825 | 0.94175 | rs13082711 | DBP |
| rs7698460 | 0.71683/0.77371 | rs13143871/rs13139571 | SBP/DBP |
| rs1077393 | 0.94047/0.79536 | rs2077492/rs805303 | DBP/SBP |
| rs4722680 | 0.84879 | rs11563582 | SBP/DBP |

**Table S10. Conjunctional cFDR: pleiotropic loci of discovery analysis in CAD and DBP (ccFDR < 0.05)**

| **RSID** | **ROLE** | **GENE** | **CHR** | **BP** | **A1** | **A2** | **P.valueA** | **P.valueB** | **ccFDR** |
| --- | --- | --- | --- | --- | --- | --- | --- | --- | --- |
| rs11066301 | intronic | *PTPN11* | chr12 | 112871372 | A | G | 5.20E-07 | 6.59E-11 | 1.04×10^-6^ |
| rs10744777 | intronic | *ALDH2* | chr12 | 112233018 | T | C | 1.52E-06 | 6.62E-07 | 4.56×10^-6^ |
| rs10774625 | intronic | *ATXN2* | chr12 | 111910219 | A | G | 7.19E-06 | 4.31E-14 | 7.19×10^-6^ |
| rs7970490 | intronic | *CUX2* | chr12 | 111756438 | A | G | 2.18E-05 | 1.48E-07 | 2.91×10^-5^ |
| rs4767293 | intergenic | *ERP29, NAA25* | chr12 | 112463296 | A | G | 1.81E-05 | 1.52E-06 | 4.07×10^-5^ |
| rs7698460 | intergenic | *GUCY1A3, GUCY1B3* | chr4 | 156666219 | G | A | 0.0010278 | 5.02E-06 | 2.06×10^-3^ |
| rs11066322 | intronic | *PTPN11* | chr12 | 112922529 | G | A | 0.0017581 | 8.15E-06 | 3.52×10^-3^ |
| rs7902587 | intergenic | *OBFC1, SLK* | chr10 | 105694301 | C | T | 7.07E-05 | 0.00185 | 1.39×10^-2^ |
| rs1077393 | intronic | *BAG6* | chr6 | 31610529 | A | G | 0.0005174 | 0.000562 | 1.67×10^-2^ |
| rs17514846 | intronic | *FURIN* | chr15 | 91416550 | C | A | 2.37E-05 | 0.00432 | 2.09×10^-2^ |
| rs10965212 | ncRNA_intronic | *CDKN2B-AS1* | chr9 | 22023795 | T | A | 1.37E-17 | 0.0219 | 2.19×10^-2^ |
| rs6489979 | intronic | *CUX2* | chr12 | 111614736 | T | C | 0.0001713 | 0.00203 | 2.32×10^-2^ |
| rs4678408 | intergenic | *NME9, MRAS* | chr3 | 138053187 | A | G | 0.0004686 | 0.001 | 2.77×10^-2^ |
| rs16824790 | intergenic | *TEX41, PABPC1P2* | chr2 | 146106518 | T | C | 6.97E-05 | 0.00534 | 3.36×10^-2^ |
| rs7069531 | intronic | *CACNB2* | chr10 | 18683267 | A | G | 0.0021776 | 0.000721 | 3.76×10^-2^ |
| rs4722680 | intergenic | *EVX1, HIBADH* | chr7 | 27319814 | A | T | 0.0218208 | 5.48E-06 | 4.05×10^-2^ |

**Note:** All loci with SNPs with ccFDR < 0.05 were used to define the number of the loci. The pleiotropic loci of CAD and DBP are presented in the above table.

**RSID:** SNP ID (rs number), **CHR:** chromosome, **BP:** base pair, **P value A:** p value of coronary artery disease, **P value B:** p value of diastolic blood pressure, **ccFDR:** Conjunctional conditional false discovery rate.

**Table S11. Conjunctional cFDR: pleiotropic loci of discovery analysis in CAD and SBP (ccFDR < 0.05)**

| **RSID** | **ROLE** | **GENE** | **CHR** | **BP** | **A1** | **A2** | **P.valueA** | **P.valueB** | **ccFDR** |
| --- | --- | --- | --- | --- | --- | --- | --- | --- | --- |
| rs11066301 | intronic | *PTPN11* | chr12 | 112871372 | A | G | 5.20E-07 | 4.94E-08 | 1.04×10^-6^ |
| rs10774625 | intronic | *ATXN2* | chr12 | 111910219 | A | G | 7.19E-06 | 1.13E-09 | 7.19×10^-6^ |
| rs10744777 | intronic | *ALDH2* | chr12 | 112233018 | T | C | 1.52E-06 | 6.24E-06 | 3.43×10^-5^ |
| rs4767293 | intergenic | *ERP29, NAA25* | chr12 | 112463296 | A | G | 1.81E-05 | 7.98E-06 | 8.15×10^-5^ |
| rs17514846 | intronic | *FURIN* | chr15 | 91416550 | C | A | 2.37E-05 | 1.17E-05 | 9.01×10^-5^ |
| rs7970490 | intronic | *CUX2* | chr12 | 111756438 | A | G | 2.18E-05 | 2.83E-05 | 1.58×10^-4^ |
| rs1077393 | intronic | *BAG6* | chr6 | 31610529 | A | G | 0.0005174 | 2.22E-06 | 1.38×10^-3^ |
| rs4243111 | intergenic | *BCAR1, CFDP1* | chr16 | 75320827 | C | T | 9.27E-05 | 0.000226 | 1.87×10^-3^ |
| rs2812 | UTR3 | *PECAM1* | chr17 | 62401118 | T | C | 0.0004253 | 7.43E-05 | 2.31×10^-3^ |
| rs805293 | intronic | *LY6G6C* | chr6 | 31688518 | T | A | 0.0004996 | 0.000109 | 2.75×10^-3^ |
| rs9515203 | intronic | *COL4A2* | chr13 | 111049623 | T | C | 3.42E-05 | 0.00145 | 6.63×10^-3^ |
| rs11066322 | intronic | *PTPN11* | chr12 | 112922529 | G | A | 0.0017581 | 0.000225 | 1.13×10^-2^ |
| rs7698460 | intergenic | *GUCY1A3, GUCY1B3* | chr4 | 156666219 | G | A | 0.0010278 | 0.000509 | 1.26×10^-2^ |
| rs7902587 | intergenic | *OBFC1, SLK* | chr10 | 105694301 | C | T | 7.07E-05 | 0.00225 | 1.27×10^-2^ |
| rs6713510 | ncRNA_intronic | *LOC646736* | chr2 | 227034499 | G | A | 9.77E-05 | 0.00544 | 3.32×10^-2^ |
| rs13154066 | intergenic | *NPR3, LOC340113* | chr5 | 32831670 | T | C | 0.0269392 | 2.12E-07 | 3.59×10^-2^ |
| rs998584 | intergenic | *VEGFA, LINC01512* | chr6 | 43757896 | C | A | 0.009024 | 9.30E-05 | 4.11×10^-2^ |
| rs13070927 | intronic | *FGD5* | chr3 | 14919646 | G | T | 0.0099472 | 0.000113 | 4.16×10^-2^ |
| rs366590 | intronic | *PLEKHA7* | chr11 | 16872440 | G | A | 0.0160067 | 3.78E-06 | 4.40×10^-2^ |

**Note:** All loci with SNPs with ccFDR < 0.05 were used to define the number of the loci. The pleiotropic loci of CAD and SBP are presented in the above table.

**RSID:** SNP ID (rs number), **CHR:** chromosome, **BP:** base pair, **P value A:** p value of coronary artery disease, **P value B:** p value of systolic blood pressure, **ccFDR:** Conjunctional conditional false discovery rate.

**Table S12. Conditional FDR value for CAD loci of replication analysis (cFDR < 0.05)**

| **RSID** | **ROLE** | **GENE** | **CHR** | **P.valueA** | **P.valueB** | **cFDR.AcB** |
| --- | --- | --- | --- | --- | --- | --- |
| rs657420 | upstream | PSRC1 | chr1 | 2.46E-05 | 1.32E-02 | 1.54E-02 |
| rs2343550 | intronic | TSPAN14 | chr10 | 5.42E-05 | 5.49E-02 | 4.48E-02 |
| rs93139 | intronic | SWAP70 | chr11 | 5.42E-05 | 2.65E-04 | 3.14E-03 |
| rs653178 | intronic | ATXN2 | chr12 | 4.04E-04 | 1.64E-14 | 4.04E-04 |
| rs7970490 | intronic | CUX2 | chr12 | 7.70E-04 | 1.48E-07 | 1.15E-03 |
| rs4767293 | intergenic | ERP29, NAA25 | chr12 | 1.36E-02 | 1.52E-06 | 2.71E-02 |
| rs4942925 | intergenic | PDS5B, LINC00423 | chr13 | 1.68E-04 | 7.80E-03 | 3.12E-02 |
| rs17514846 | intronic | FURIN | chr15 | 2.22E-04 | 1.17E-05 | 2.44E-03 |
| rs7584363 | intronic | CERS6 | chr2 | 4.20E-05 | 3.94E-02 | 3.73E-02 |
| rs1887320 | intergenic | LOC101929413, C20orf187 | chr20 | 1.67E-03 | 9.94E-07 | 2.78E-03 |
| rs10517621 | intergenic | GUCY1A3, GUCY1B3 | chr4 | 1.86E-03 | 1.22E-05 | 5.59E-03 |
| rs3892368 | intronic | NWD2 | chr4 | 1.23E-05 | 6.37E-02 | 2.38E-02 |
| rs491645 | intergenic | LINC02516, ANKRD50 | chr4 | 2.68E-04 | 6.16E-04 | 8.67E-03 |
| rs17083481 | intergenic | LOC100506444, RPL21P44 | chr4 | 1.26E-05 | 1.23E-02 | 1.48E-02 |
| rs3776307 | intronic | ARHGAP26 | chr5 | 7.76E-05 | 7.05E-03 | 2.65E-02 |
| rs2876303 | intronic | PHACTR1 | chr6 | 2.10E-10 | 2.11E-02 | 2.06E-07 |
| rs9381462 | intronic | PHACTR1 | chr6 | 2.37E-07 | 1.53E-01 | 1.65E-03 |
| rs7454215 | intergenic | THBS2, WDR27 | chr6 | 1.30E-03 | 1.09E-03 | 3.66E-02 |
| rs11765576 | intergenic | PODXL, LOC101928782 | chr7 | 1.32E-04 | 7.21E-03 | 3.08E-02 |
| rs7049105 | ncRNA_intronic | CDKN2B-AS1 | chr9 | 9.28E-14 | 2.07E-02 | 1.79E-10 |
| rs1333050 | intergenic | CDKN2B-AS1, DMRTA1 | chr9 | 8.67E-15 | 3.58E-01 | 2.86E-10 |

**RSID:** SNP ID (rs number), **CHR:** chromosome, **P value A:** P-value of coronary artery disease, **P value B:** P-value of systolic blood pressure or diastolic blood pressure, **cFDR:** conditional false discovery rate.

**Table S13. Conditional FDR value for BP loci of replication analysis (cFDR < 0.05)**

| **RSID** | **ROLE** | **GENE** | **CHR** | **P.valueA** | **P.valueB** | **cFDR.BcA** |
| --- | --- | --- | --- | --- | --- | --- |
| rs12562952 | intergenic | NPPB, KIAA2013 | chr1 | 8.20E-02 | 3.31E-05 | 3.66E-02 |
| rs2588917 | intronic | C10orf107 | chr10 | 9.83E-01 | 2.43E-07 | 5.62E-03 |
| rs10786152 | intronic | PLCE1 | chr10 | 2.86E-01 | 1.53E-06 | 1.10E-02 |
| rs93139 | intronic | SWAP70 | chr11 | 5.42E-05 | 2.65E-04 | 5.30E-03 |
| rs7108385 | ncRNA_intronic | CAND1.11 | chr11 | 9.13E-01 | 1.90E-06 | 3.28E-02 |
| rs653178 | intronic | ATXN2 | chr12 | 4.04E-04 | 1.64E-14 | 1.20E-12 |
| rs7970490 | intronic | CUX2 | chr12 | 7.70E-04 | 1.48E-07 | 9.03E-06 |
| rs4767293 | intergenic | ERP29, NAA25 | chr12 | 1.36E-02 | 1.52E-06 | 6.20E-04 |
| rs2384550 | intergenic | TBX3, MED13L | chr12 | 9.24E-01 | 1.01E-06 | 1.76E-02 |
| rs1327620 | intergenic | KLF5, LINC00392 | chr13 | 5.61E-03 | 2.32E-04 | 3.42E-02 |
| rs4886417 | intronic | PPCDC | chr15 | 6.27E-03 | 2.90E-04 | 3.92E-02 |
| rs17514846 | intronic | FURIN | chr15 | 2.22E-04 | 1.17E-05 | 5.27E-04 |
| rs11070252 | intergenic | TJP1, GOLGA8J | chr15 | 2.84E-01 | 5.74E-06 | 2.73E-02 |
| rs747039 | intronic | ZNF652 | chr17 | 1.22E-02 | 6.83E-05 | 2.02E-02 |
| rs8089281 | intergenic | TSHZ1, SMIM21 | chr18 | 7.27E-03 | 2.76E-04 | 4.25E-02 |
| rs10865186 | intergenic | HAAO, LINC01819 | chr2 | 1.33E-01 | 2.70E-06 | 5.35E-03 |
| rs1887320 | intergenic | LOC101929413, C20orf187 | chr20 | 1.67E-03 | 9.94E-07 | 8.02E-05 |
| rs4245909 | ncRNA_intronic | LOC105374205 | chr3 | 5.31E-02 | 1.22E-06 | 1.79E-03 |
| rs17284933 | intergenic | ULK4, TRAK1 | chr3 | 2.57E-01 | 1.55E-06 | 5.73E-03 |
| rs11130138 | intergenic | ELP6, CSPG5 | chr3 | 8.82E-03 | 1.44E-04 | 3.18E-02 |
| rs10517621 | intergenic | GUCY1A3, GUCY1B3 | chr4 | 1.86E-03 | 1.22E-05 | 8.24E-04 |
| rs491645 | intergenic | LINC02516, ANKRD50 | chr4 | 2.68E-04 | 6.16E-04 | 1.07E-02 |
| rs13154066 | intergenic | NPR3, LINC02120 | chr5 | 1.13E-01 | 2.12E-07 | 1.26E-03 |
| rs12201798 | intergenic | RFX6, VGLL2 | chr6 | 6.14E-01 | 2.04E-06 | 1.51E-02 |
| rs2876303 | intronic | PHACTR1 | chr6 | 2.10E-10 | 2.11E-02 | 3.17E-02 |
| rs1077393 | intronic | BAG6 | chr6 | 1.36E-01 | 2.22E-06 | 7.85E-03 |
| rs9467445 | intergenic | CMAHP, LOC101928663 | chr6 | 5.87E-01 | 3.33E-06 | 2.70E-02 |
| rs7454215 | intergenic | THBS2, WDR27 | chr6 | 1.30E-03 | 1.09E-03 | 3.54E-02 |
| rs2969070 | intergenic | LOC101927181, GRIFIN | chr7 | 6.39E-02 | 2.57E-09 | 9.00E-06 |
| rs2051924 | intergenic | EVX1, HIBADH | chr7 | 1.40E-01 | 1.33E-05 | 2.16E-02 |
| rs7049105 | ncRNA_intronic | CDKN2B-AS1 | chr9 | 9.28E-14 | 2.07E-02 | 4.14E-02 |

**RSID:** SNP ID (rs number), **CHR:** chromosome, **P value A:** P-value of coronary artery disease, **P value B:** P-value of systolic blood pressure or diastolic blood pressure, **cFDR:** conditional false discovery rate.

**Table S14. Conjunctional cFDR: pleiotropic loci in CAD and BP of replication analysis (ccFDR < 0.05)**

| **RSID** | **ROLE** | **GENE** | **CHR** | **P.valueA** | **P.valueB** | **ccFDR** |
| --- | --- | --- | --- | --- | --- | --- |
| rs93139 | intronic | SWAP70 | chr11 | 5.42E-05 | 2.65E-04 | 5.30E-03 |
| rs7970490 | intronic | CUX2 | chr12 | 7.70E-04 | 1.48E-07 | 1.15E-03 |
| rs4767293 | intergenic | ERP29, NAA25 | chr12 | 1.36E-02 | 1.52E-06 | 2.71E-02 |
| rs653178 | intronic | ATXN2 | chr12 | 4.04E-04 | 9.30E-10 | 4.04E-04 |
| rs17514846 | intronic | FURIN | chr15 | 2.22E-04 | 1.17E-05 | 2.44E-03 |
| rs1887320 | intergenic | LOC101929413, C20orf187 | chr20 | 1.67E-03 | 9.94E-07 | 2.78E-03 |
| rs10517621 | intergenic | GUCY1A3, GUCY1B3 | chr4 | 1.86E-03 | 1.22E-05 | 5.59E-03 |
| rs491645 | intergenic | LINC02516, ANKRD50 | chr4 | 2.68E-04 | 6.16E-04 | 1.07E-02 |
| rs2876303 | intronic | PHACTR1 | chr6 | 2.10E-10 | 2.11E-02 | 3.17E-02 |
| rs7454215 | intergenic | THBS2, WDR27 | chr6 | 1.30E-03 | 1.09E-03 | 3.66E-02 |
| rs7049105 | ncRNA_intronic | CDKN2B-AS1 | chr9 | 9.28E-14 | 2.07E-02 | 4.14E-02 |

**RSID:** SNP ID (rs number), **CHR:** chromosome, **P value A:** P-value of coronary artery disease, **P value B:** P-value of systolic blood pressure or diastolic blood pressure, **ccFDR:** Conjunctional conditional false discovery rate.


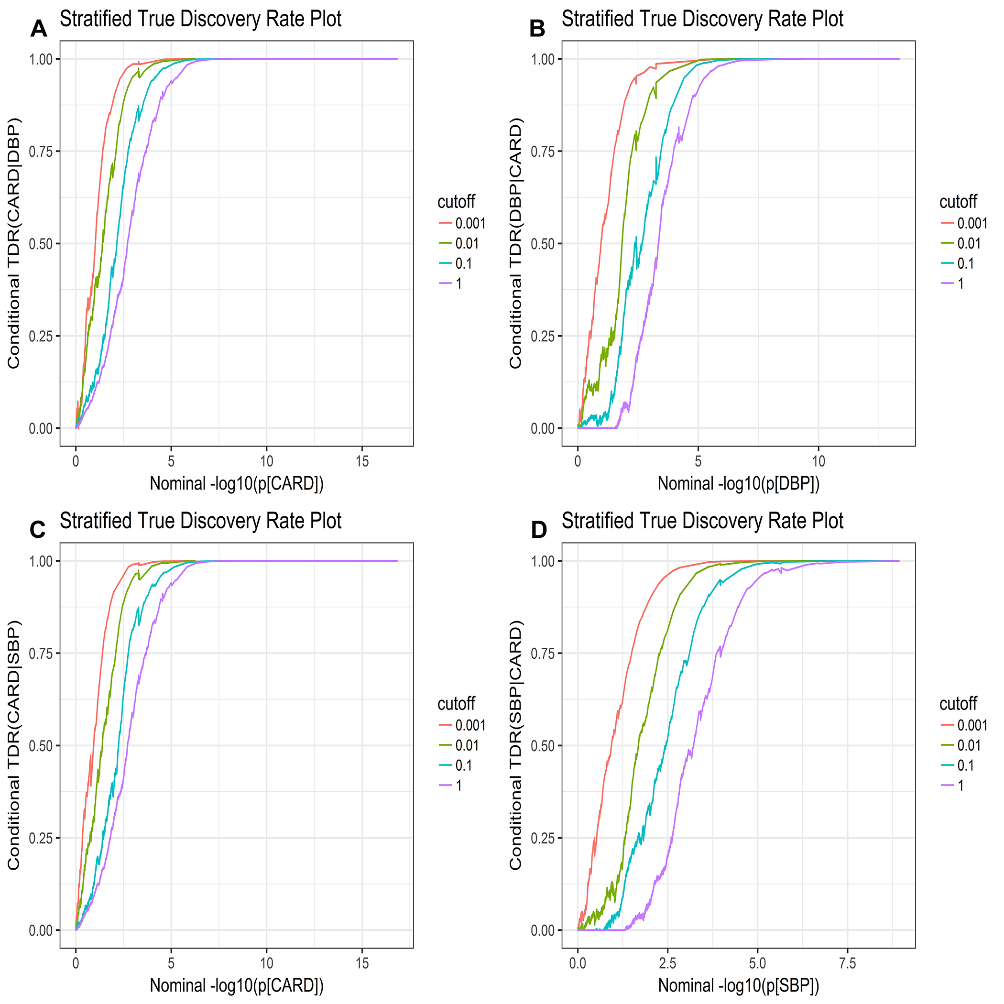


**Figure S1 Stratified True Discovery Rate (TDR) plots** illustrating the increase in TDR associated with increased pleiotropic enrichment in **A)** nominal CAD *p*-values conditional on DBP (CAD|DBP), and in **B)** nominal DBP *p*-values conditional on CAD (DBP|CAD), and in **C)** CAD conditional on SBP (CAD|SBP), and **D)** nominal SBP *p*-values conditional on CAD (SBP|CAD).

**
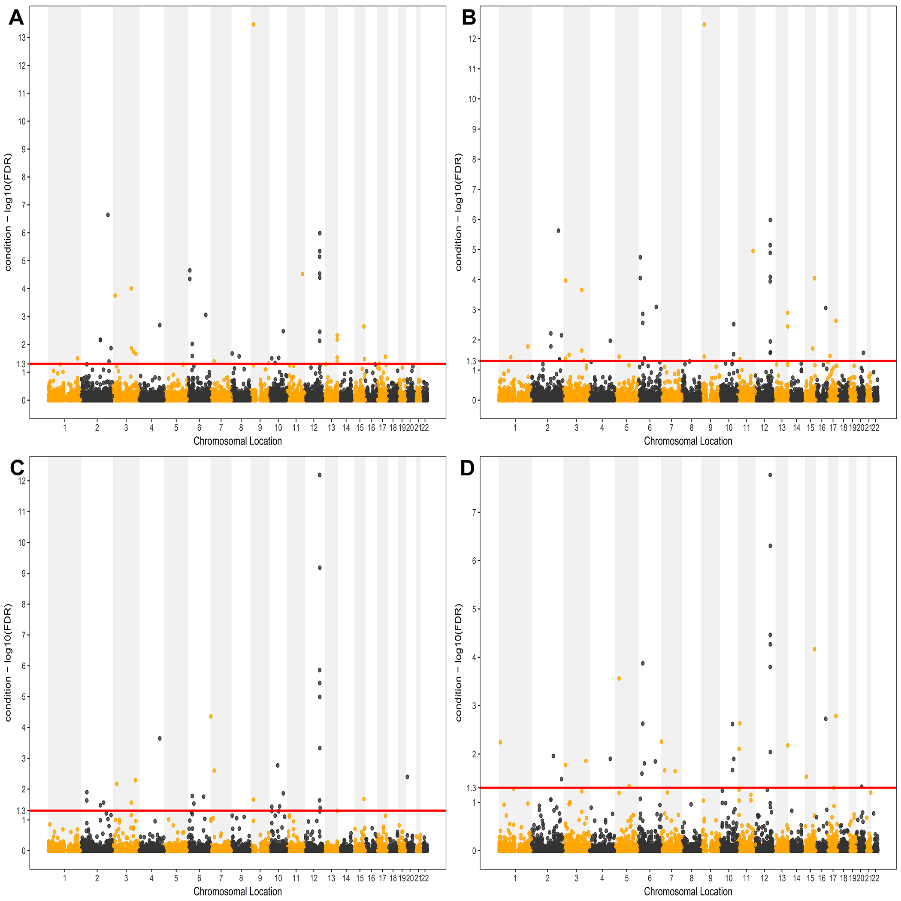
**

**Figure S2** **“Conditional Manhattan Plot” of -log_10_ (cFDR) values for CAD and BP in discovery analysis. A:** CAD conditioned on DBP, SNPs with -log_10_ cFDR＞1.3 (i.e. cFDR＜0.05) are shown above the red line and 42 SNPs with -log_10_ (cFDR) values greater than the threshold value. The small points above the red line mark the chromosomal locations of significant loci. Further details about loci are offered in Table S2. **B:** CAD conditioned on SBP, 44 SNPs with -log cFDR values greater than 1.3. Further details are offered in Table S3. **C:** DBP conditioned on CAD, 28 SNPs with -log cFDR values greater than 1.3**.** Further details are offered in Table S6. **D:** SBP conditioned on CAD**,** 33 SNPs with -log cFDR values greater than 1.3. Further details are offered in Table S7.


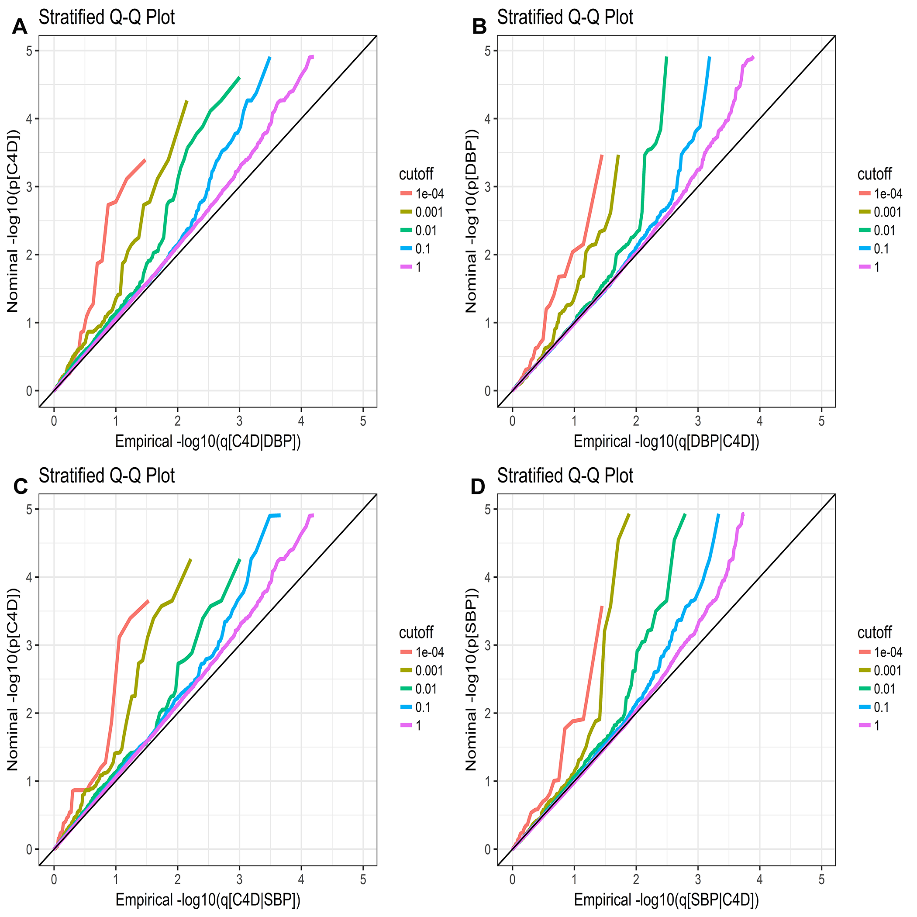


**Figure S3 Stratified Q-Q plots of replication analysis for CAD and BP.** The plots showed the pleiotropic enrichment pattern replicated in the C4D GWAS dataset.
